# Effects of Antioxidant Gene Overexpression on Stress Resistance and Malignization In Vitro and In Vivo: A Review

**DOI:** 10.3390/antiox11122316

**Published:** 2022-11-23

**Authors:** Marina M. Tavleeva, Elena S. Belykh, Anna V. Rybak, Elena E. Rasova, Aleksey A. Chernykh, Zaur B. Ismailov, Ilya O. Velegzhaninov

**Affiliations:** 1 Institute of Biology of Komi Scientific Centre, Ural Branch of Russian Academy of Sciences, 28b Kommunisticheskaya St., Syktyvkar 167982, Russia; 2 Institute of Physiology of Komi Scientific Centre, Ural Branch of Russian Academy of Sciences, 50 Pervomaiskaya St., Syktyvkar 167982, Russia

**Keywords:** antioxidant genes, overexpression, stress resistance, carcinogenesis, oncosuppression, gene therapy

## Abstract

Reactive oxygen species (ROS) are normal products of a number of biochemical reactions and are important signaling molecules. However, at the same time, they are toxic to cells and have to be strictly regulated by their antioxidant systems. The etiology and pathogenesis of many diseases are associated with increased ROS levels, and many external stress factors directly or indirectly cause oxidative stress in cells. Within this context, the overexpression of genes encoding the proteins in antioxidant systems seems to have become a viable approach to decrease the oxidative stress caused by pathological conditions and to increase cellular stress resistance. However, such manipulations unavoidably lead to side effects, the most dangerous of which is an increased probability of healthy tissue malignization or increased tumor aggression. The aims of the present review were to collect and systematize the results of studies devoted to the effects resulting from the overexpression of antioxidant system genes on stress resistance and carcinogenesis in vitro and in vivo. In most cases, the overexpression of these genes was shown to increase cell and organism resistances to factors that induce oxidative and genotoxic stress but to also have different effects on cancer initiation and promotion. The last fact greatly limits perspectives of such manipulations in practice. The overexpression of *GPX3* and *SOD3* encoding secreted proteins seems to be the “safest” among the genes that can increase cell resistance to oxidative stress. High efficiency and safety potential can also be found for *SOD2* overexpression in combinations with *GPX1* or *CAT* and for similar combinations that lead to no significant changes in H_2_O_2_ levels. Accumulation, systematization, and the integral analysis of data on antioxidant gene overexpression effects can help to develop approaches for practical uses in biomedical and agricultural areas. Additionally, a number of factors such as genetic and functional context, cell and tissue type, differences in the function of transcripts of one and the same gene, regulatory interactions, and additional functions should be taken into account.

## 1. Introduction

Reactive oxygen species (ROS) participate in the regulation of physiological processes, including gene expression regulation, the activation of stress-response signaling cascades, and apoptosis (for a review, see [1]). Mitochondria, which are the main source of ROS in the cell, use them as signal molecules [2]. At submicromolar concentrations, ROS induce the proliferative activity of cells, working as mitogens. It is known [3] that mitochondria can activate cellular immune response by changing their membrane potential and by directly interacting with Toll-like receptors as well as by participating in signaling that regulates innate immune responses. However, ROS can cause macromolecule damage [1], and therefore, their levels are strictly regulated by antioxidant systems [4].

Oxidative stress is a disbalance between ROS and reactive nitrogen species (RNS) on the one hand and antioxidant defense on the other. Among the causes of oxidative stress are the imperfect Red-Ox regulation of normal physiological processes, primarily anaerobic respiration, and oxidative phosphorylation in mitochondria [5], as well as decreased antioxidant protein levels due to pathological conditions or senescence. Oxidative stress can be the result of chemical and physical factors with direct ROS and RNS generation activity or can be achieved indirectly by acting through endocrine disruption [6,7,8]. Causality works both ways when it comes to the relationship between oxidative stress and inflammation. Through various signaling cascades, chronic oxidative stress can activate pro-inflammatory cytokines [9], and the inflammation itself, in turn, leads to the production of additional amounts of ROS [10]. The emerging vicious circle underlies many pathologies, including the initiation of carcinogenesis [9,11].

Oxidative stress is an important component in the etiology and pathogenesis of oncology [5,12], cardiovascular (ischemia-reperfusion) diseases [13], neurodegenerative diseases (Alzheimer’s, Parkinson’s diseases, etc.) [12,14,15], epilepsy [16], Huntington’s disease [17,18], diabetes mellitus [19], and multiple organ dysfunction syndrome [20]. In addition to that, oxidative stress is associated with mechanisms of psoriasis development [21,22,23,24] and with skin fibrosis in systemic scleroderma [25]. Oxidative stress, which is primarily caused by mitochondrial dysfunction, plays one of the leading roles in the processes of aging, both in tissues and in the whole organism [5,26,27]. All that makes our knowledge of the possibilities and consequences of cell antioxidant systems regulation very important for the development of strategies and approaches for reducing the negative effects of oxidative stress as well as for prophylaxis and the treatment of a wide variety of diseases. The main strategies can be classified into pharmacological, nutritiological, genetic, and ecological [28,29,30,31]. The latter is more concerned with preventing the formation of ROS where possible [28]. Naturally, the most straightforward strategies are built on pharmacological interventions, predominantly with various antioxidants. Studies on the topic include assessments of the effects of curcumin and curcuminoids [32,33], plant-derived polyphenols [34,35], flavonoids [36], boswellic acids [37], coenzyme Q10 [38], quercetin [39] N-acetylcysteine [40], monoclonal antibodies to cytokines (infliximab, tocilizumab [41]), and many other compounds and pharmaceuticals. The efficacy and success rates of pharmacological strategies are, as one can conclude, limited, with four possible reasons suggested: (1) vitamin antioxidants based upon in vitro studies may be inefficient scavengers in vivo; (2) detrimental ROS may be compartmentalized at sites within the cell that are inaccessible by the administered antioxidant; (3) antioxidants may possess toxicities that mask their advantageous effect; and (4) heterogeneity of the groups under study [42]. The use of nutritional approaches to treat and prevent diseases and to leveling unhealthy environment adverse effects comes from eating foods rich in antioxidants or food additives/drugs based on such foods [43,44]. However, systematic or meta-analysis results suggest that eating antioxidants is more often harmless, but there is insufficient evidence on the positive effects of this for disease treatment or for the improvement of life quality [43,45,46], or the benefits are only revealed under a strictly defined set of measures or under a strictly defined disease stage with the observance of dosages [47,48,49,50]. Genetic approaches involve the regulation of the cell’s own antioxidant system. The results obtained when discovering the targets for such regulation are reviewed below.

In addition, the rational control of antioxidant system activity is required for the regulation of organisms and for normal cells’ resistance to stressful and adverse conditions. It is also required to regulate the resistances of malignant cells and tissues to chemotherapeutics and irradiation. Previously, for example, gene therapies with constructs that transiently overexpress antioxidant genes were used to elucidate approaches for protecting normal tissues during tumor radiotherapy [51]. Taking into account that almost all intracellular systems, including antioxidant ones, consist of more than one protein, approaches utilizing transcription regulation of antioxidant genes in functionally rational combinations seem to be the most promising ones [52]. These approaches have become viable because of our vast knowledge of the functions of both individual-proteins and whole-protein subsystems and because of development of CRISPRa and CRISPRi technologies for the regulation of gene expression in cells. At the same time, one must take into account potential side effects. For example, the overexpression of a transcription factor gene *NFE2L2* (also known as *Nrf2*) can lead to the induction of skin anomalies in mice [53], and the overexpression of *SOD1* without the simultaneous overexpression of H_2_O_2_-utilizing enzymes genes lead to the faster loss of function in retina cones. *GPX4* overexpression decreased these negative effects of *SOD1* overexpression [54]. However, the most important finding was that the overexpression of particular antioxidant genes could increase or decrease the probability of malignization as well as differently affect processes in already-developed tumor tissues.

The aim of this review was to systematize the results of gain of function studies of antioxidant systems genes and to search for candidate genes and their combinations for the safe and effective treatment and prevention of oxidative stress with different genesis. Special attention was devoted to the results of experimental studies covering the effects resulting from the overexpression of particular antioxidant genes and their combinations on the stress resistance of cells, the probability of tumor induction, and tumor progression. We believe that systematization would be an important intermediate point for further studies for the manipulation and control of oxidative stress resistance in cells and organisms.

## 2. Loss of Function Studies of Antioxidant Genes

Obviously, knockout or knockdown genes, products of which are components of primary life-support systems ([55] for review), result in the decreased viability and stress resistance of cells and organisms and an increased probability of malignization in the majority of cases.

For example, the knockdown or knockout of antioxidant defense genes decreased cell resistance to pro-oxidants (*SOD2*: [56]; *SOD1*, *TRX1*: [57]; *PRXD2*: [58]; *PRDX1*: [59]; *CAT*: [60]; *PRDX2*: [61]), alkylating agents (*PRDX1*: [62]; *PRDX3*: [63]; *PRDX2*: [64]), and ionizing radiation (*PRDX1*: [65]; *PRDX4*: [66]; *PRDX1*, *PRDX5*: [67]; *GSL2*: [68]; *PRDX2*: [61,64]). At the scale of the whole organism, *PRDX1* knockdown in mice led to decreased lifespan, the development of hemolytic anemia, and malignant neoplasms (lymphomas, sarcomas, and carcinomas) [59]. The suppression of *NFE2L2* significantly affected the development of endothelial dysfunction and decreased microvascular bed density in normotensive rats under oxidative stress caused by NaCl [69]. Decreased *SOD1* activity elevated the neurotoxic potential of quinolonic and kainic acids in the corpus striatum of transgenic mice [70].

However, there are opposite examples of the effects of antioxidant gene knockout, knockdown, or suppression. *TRXR1* knockdown in A549 cells decreased their resistance to 1-chloro-2,4-dinitrobenzene or menadione, but at the same time, it significantly increased their resistance to cisplatin. This effect can be explained by TrxR1 protein derivatized with cisplatin being able to induce cell death [71].

The results of a number of loss of function studies were very important for deciphering the functions of many antioxidant genes and of the mechanisms of antioxidant systems. Presently, the elements of the reaction cascade that provides Red/Ox homeostasis are well-studied. These data are required for the development of new approaches to overcome tumor resistance to chemo- and radiotherapy as well as to decrease side effects in normal tissues and whole organisms [1,51]. However, much less is known about such effects on the cellular level and at the organizational levels above it that are induced by the increased gene expression of antioxidant proteins, both individually and in various combinations. Although such interventions also interfere with a fine-tuned cell balance, the effects produced are significantly diverse, and not only bring new fundamental knowledge, but also have potential importance in applied contexts. Therefore, the systematic review we present here is focused on the available results of gain of function studies of antioxidant defense genes.

## 3. Effects of Antioxidant Defense Gene Overexpression on Stress Resistance

The main aim of this review was to systemize and sum up the available studies covering the effects of antioxidant defense gene overexpression on cell resistance to oxidative stress. We analyzed 166 such works [20,51,56,58,62,65,66,71,72,73,74,75,76,77,78,79,80,81,82,83,84,85,86,87,88,89,90,91,92,93,94,95,96,97,98,99,100,101,102,103,104,105,106,107,108,109,110,111,112,113,114,115,116,117,118,119,120,121,122,123,124,125,126,127,128,129,130,131,132,133,134,135,136,137,138,139,140,141,142,143,144,145,146,147,148,149,150,151,152,153,154,155,156,157,158,159,160,161,162,163,164,165,166,167,168,169,170,171,172,173,174,175,176,177,178,179,180,181,182,183,184,185,186,187,188,189,190,191,192,193,194,195,196,197,198,199,200,201,202,203,204,205,206,207,208,209,210,211,212,213,214,215,216,217,218,219,220,221,222,223,224,225,226,227] published during the period covering 1988–2022. Table 1 contains extremely condensed information on the effects of overexpression of the following antioxidant genes (with direct and indirect antioxidant action): *SOD1*, *SOD2*, *SOD3* (*EC-SOD*), *CAT*, *MTI*, *MTII*, *ALDH3A1*, *TRX1*, *TRX2*, *GCLC*, *GCLM*, *GPX1*, *TXN1*, *NQO1*, *PRDX1*, *PRDX2*, *PRDX3*, *PRDX4*, *PRDX5*, *PRDX6*, *TXD1*, *SLC31A1*, *SLC7A11*, *GPX2*, *GPX3*, *GPX4*, *GSTP1*, *SRXN1*, *TRXR1*, *PON1*, *PON2*, and *PON3*. More detailed information about the analyzed studies is presented in the Supplementary Materials (Table S1). Resistance in vivo or in vitro was assessed to determine the oxidative stress caused by various irradiation types (UV, X-rays, and gamma), chemical compounds (hydrogen peroxide, paraquat, etc.), and other factors (ischemia, sepsis, surgical interventions, hypoxia, and endotoxemia). In the majority of published studies that we analyzed, the overexpression of antioxidant defense genes in vitro and in vivo was achieved by introducing an additional copy of the gene or a transgene. CRISPRa technology, which became available in 2013 [228], despite its obvious advantages [52], was registered in one work [177].

Cases of spontaneous gene overexpression in tumors resistant to particular therapy types were also reviewed, but only in such cases where the abolition of overexpression of a particular gene led to the abolition of the changed phenotype.

In most of the studies analyzed, the overexpression of antioxidant defense genes resulted in increased resistance to oxidative stress induced by different factors, both in vitro and in vivo. For example, the increased survival of cells was found in NK-92 and NK-92MI exposed to H_2_O_2_ (*PRDX1*, [151]). Mice overexpressing *TRX1* survived better after adriamycin treatment [219], and mice overexpressing *PON1* or *PON3* had less liver damage after treatment with carbon tetrachloride [135]. Decreased apoptosis levels were observed for *PRDX6* in vitro in SCOV-3 cell lines treated with cisplatin [169], for *PRDX5* in human tendon fibroblasts treated with H_2_O_2_ [161], and in HT29 cells treated with shikonin [166], for *TRX1* in WEHI7.2 lymphoma cells treated with H_2_O_2_ [220], in in vivo studies on transgenic mice that were treated with methamphetamine [214], and for *SRXN1* in retinal ganglion cells (RGC) of mice treated with high glucose concentrations [229]. Among the positive effects of overexpression, for *PRDX5*, researchers have shown decreased DNA damage in hamster cell cultures CHO-K1 exposed to H_2_O_2_ and tert-butylhydroperoxide (tBHP) [162] as well as for *TRX1* in the mitochondria of transgenic mice Trx1-Tg in vivo in sepsis-induced myocardial dysfunction and sham surgery [216,217]. Increased expression of *TRX1* in mice in vivo [217] and ex vivo [218] decreased the negative consequences of ischemia for the cardiovascular system. Using oxygen–glucose deprivation/reoxygenation in in vitro experiments showed that the ability of *PON2* to enhance glucose transport and to suppress oxidative stress and apoptosis is potentially a good set of properties to prevent cerebral ischemia-reperfusion injury [143].

In some cases, the authors noted that increased expression of the antioxidant defense genes in fact decreases cell resistance to oxidative stress; however, such studies are scarce. For example, decreased resistance to oxidative stress was shown in the brain neurons of mice overexpressing *SOD1* when exposed to menadione [182], in human cells overexpressing *NFE2L2* that were treated with cisplatin [126,129], and in human cells overexpressing *GPX1* when exposed to radiation [100]. No changes in stress resistance were found in human cells TK6 overexpressins *SOD1* when treated with gamma-irradiation [181]; in normal human keratinocytes overexpressing *SOD1* when irradiated with UV [73]; in brain neurons of mice overexpressing *SOD1* treated with H_2_O_2_ [182]; in Chinese hamster ovary cells overexpressing *MTII* when treated with gamma-rays, bleomycin, MMS, and N-hydroxyethyl-N-chloroethylnitrosourea [122]; in mouse C127 cells overexpressing *MTII* that had been exposed to 5-fluorouracil and vincristine [123]; in Chinese hamster cells V79 overexpressing *MTI* when exposed to alkylating agents [121]; in vivo in *D. melanogaster* overexpressing *SOD1* when the flies were exposed to paraquat [185]; and in *SOD2*-overexpressing ones exposed to 100% O_2_ [199].

The number of studies that have shown the overexpression of various antioxidant defense genes increasing the oxidative stress resistance on the cell and organism levels gives ground to careful optimism when assessing the future feasibility of gene expression regulation in these genes for therapeutic purposes. However, the in vitro effects that are considered beneficial at that level might be dangerous or even harmful at higher levels of structural and functional organization. Similarly, relatively positive effects in in vivo models that have been observed to be beneficial at the endpoints selected by researchers might cause negative side effects in the long term according to other parameters. Taking this into account, an extra task was to assess the effects of the increased expression of the antioxidant defense genes on the probabilities of malignization in normal cells and on the growth and development of tumor cells.

## 4. Effects of the Antioxidant Defense Genes Overexpression on Carcinogenesis

**Figure 1:**
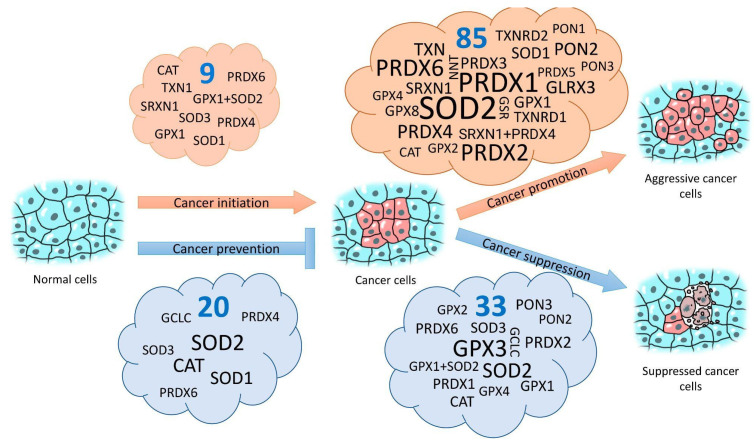
Effects of the overexpression of antioxidant defense genes on the probability of normal cells/tissues malignization and on the aggressiveness of already developed cancer cells/tumors. The font size is proportional to the number of studies that showed the effect of the overexpression of that gene. Numbers in blue show total numbers of studies in each group.

Logically, the overexpression of antioxidant genes, as said above, has predominantly cytoprotective effects and must decrease the frequency of spontaneous and induced mutations and therefore must decrease the probability of malignization. On the other hand, in already malignantly transformed cells, it can stimulate faster proliferation, decrease apoptosis and permanent cell cycle arrest, and increase tumor resistance to therapeutic interventions. Previously, the authors of a study on *PRDX6* came to similar conclusions [230]. Another review devoted to glutathione peroxidases came to similar conclusions [231], with the authors stating that all *GPX*s prevent cancer initiation in normal cells by removing hydroperoxides, but that in already malignantly transformed cells, they have various effects, including tumor promotion. In a limited set of studies on *SOD3* overexpression, a completely opposite picture can be found. The increased expression of *SOD3* led to the immortalization of mouse embryonic fibroblasts and carcinogenesis despite the fact that, in the developed tumors, this gene’s expression is usually suppressed [232]. Additionally, there is direct evidence that *SOD3* overexpression suppresses growth and aggression in tumor cells both in vitro and in vivo [233,234]. Limited samples of focused studies of the overexpression of other genes also support and disprove the proposed simplified idea (see Figure 1 and Table S2). Additionally, we often talk about one and the same gene. For instance, increased *SOD2* expression has often been observed in tumor tissues and is frequently correlated with tumor stage and metastasis activity [235,236]. More than that, some studies have demonstrated that *SOD2* overexpression in tumors promotes growth and metastasis [237,238] and increases resistance to anoikis [239]. However, there are many works that present evidence for the opposite. Cancer cells from the MCF-7, U118, and MIA PaCa-2 lines found to overexpress *SOD2* when injected into nude mice showed significantly lesser and slower tumor development and decreased mortality when compared with the same cell lines without *SOD2* overexpression [240,241,242]. An even more pronounced effect of decreased tumor growth and increased animal survival was achieved in MIA PaCa-2 cells when *SOD2* and *GPX1* were overexpressed simultaneously [243]. The overexpression of *SOD2* with the lentiviruses that deliver this gene with powerful promotors, in human cell cultures and in xenografts, and in surrounding tissues in mice simultaneously led to the decreased resistance and proliferation of cancer cells and provided protection against radiation damage for normal cells at the same time [206]. In other types of experiments, increased expression of *SOD2* also suppressed the development and metastasis of tumors. In contrast with the results of the study mentioned above, in which increased levels of H_2_O_2_ associated with *SOD2* overexpression stimulated the migration and invasion of HT-1080 cells [238], the same effects were achieved by suppressing *SOD2* expression in ovarian cancer cells resulting from increased superoxide–radical concentrations [244,245]. In other words, in different experimental systems, *SOD2* overexpression can either stimulate tumor growth and metastasis or suppress them. It seems that an important role belongs to the ratios of expression of superoxide dismutases with expression of *CAT* and/or *GPX*s, the protein products of which have hydrogen peroxide as a substrate [236]. As a consequence, H_2_O_2_ and superoxide radicals probably are among the key signal molecules involved in the regulation of such processes [238,244,245,246,247,248].

The avoidance of H_2_O_2_ overproduction is likely to be the cause of no promotion of cancer cells when there is simultaneous overexpression of *SOD2* and *CAT* both in vitro [247] and in vivo [246] and of tumor-preventive [249] and tumor-suppressive [243] effects in the case of the simultaneous overexpression of *SOD2* and *GPX1*. This is another argument for higher efficacy antioxidant gene overexpression in functionally viable combinations in contrast with the overexpression of individual genes.

A graphical representation of the effects of the overexpression of antioxidant genes on the induction, prevention, promotion, or suppression of tumor processes (based on an analysis of 130 studies) [58,63,66,87,88,89,90,103,104,105,114,117,159,173,174,175,192,201,210,230,232,233,234,238,239,240,241,242,243,244,245,246,247,248,249,250,251,252,253,254,255,256,257,258,259,260,261,262,263,264,265,266,267,268,269,270,271,272,273,274,275,276,277,278,279,280,281,282,283,284,285,286,287,288,289,290,291,292,293,294,295,296,297,298,299,300,301,302,303,304,305,306,307,308,309,310,311,312,313,314,315,316,317,318,319,320,321,322,323,324,325,326,327,328,329,330,331,332,333,334,335,336,337,338,339,340,341,342,343] is given in Figure 1. More detailed information and references to actual studies are provided in Table S2.

## 5. Problems and Perspectives of Applied Regulation of Cell Stress Resistance by Overexpressing the Antioxidant Defense Genes

Based on the above, the overexpression of antioxidant defense genes has practical potential for the therapeutic regulation of oxidative stress and for increasing cellular stress resistance. On the other hand, studies on side effects of such overexpression, primarily its cancerogenic potential, give very contradictory results, and we have to first elucidate which genes and in which context overexpression can occur without incurring unjustified risks.

For example, *SOD2* overexpression, the consequences of which are apparently the best-studied ones, seems to be counterproductive without the simultaneous overexpression of genes, products of which eliminate H_2_O_2_, such as *CAT* or *GPXs*. Among the last group the most applicable ones are *GPX1* [243,249], the overexpression of which by itself has different effects on cancer initiation and development in different experimental systems (cancer initiation [249]; promotion [103,105,301]; and suppression [104,243]). The same is true for *GPX4* [231,340]. Catalase looks like an obvious and even more viable partner for the simultaneous overexpression with *SOD2*. Firstly, for this gene combination, we already know the effect of increased cell resistance to gamma rays [82]. Secondly, it was reported that catalase prevents *SOD2* overexpression-induced promotion of cancer cells in vitro [247] and in vivo [246]. Thirdly, the isolated overexpression of *CAT* in experiments led to the prevention of malignant transformation [87,318,319,320,321] or to the suppression of tumor cells growth [89,90] significantly more frequently than it did to cancer promotion [88]. It is possible that cytoplasmic superoxide dismutase (*SOD1*) is an even more “suitable” partner for *CAT* overexpression because for this combination, increased cell resistance to UV [73], paraquat, and H_2_O_2_ [83] were observed in vitro, and increased cell resistance to benzopyrene was observed in vivo [75]. However, data on effects of the simultaneous expression of *CAT* and *SOD1* on cancer promotion are not available.

A special attention is given to one other member of the glutathione peroxidase family s*GPX3*. Its overexpression increased cell resistance to ascorbate [109] and cisplatin [110]. Despite the fact that these findings were made in tumor cells that spontaneously overexpressed *GPX3* and that high *GPX3* expression was associated with poorer overall survival in patients with ovarian cancer and with increased tumor stage [109], all direct manipulations with *GPX3* overexpression conversely indicate the potential safety of such manipulation from the perspective of carcinogenesis. The suppression of proliferation, migration, and invasion in vitro [326,327,332,333] as well as of tumorigenicity in vivo [328,329,330,331] were shown in models overexpressing *GPX3*. Additionally, several other studies have provided evidence that in tumor tissues and cancer cell lines, *GPX3* expression is suppressed or completely blocked due to promoter hypermethylation or gene deletion [329,331,344,345,346]. Thus, GPX3 functions as a tumor suppressor in an overwhelming majority of studies, and its overexpression, isolated or in combination with other components of the antioxidant system, is very promising for the suppression of endogenous or exogenous oxidative stress.

*GPX3* encodes a secreted protein; therefore, *SOD3*, which encodes extracellular superoxide dismutase, could be a potentially effective functional partner for it. We know that transgenic mice that overexpress *SOD3* have increased resistance to rib cage irradiation with 4-MV photons [208], to focal cerebral ischemia [209], to 12-O-tetradecanoylphorbol-13-acetate (TPA) exposure [210], and to lipopolysaccharide-induced endotoxemia [20]. At the same time, *SOD3* overexpression decreases dimethylbenzanthracene/TPA-induced tumor formation [210] and suppresses cancer cells’ growth in vitro [233,234] and in vivo [234]. However, increased expression of *SOD3* in mouse embryonic fibroblasts, can induce carcinogenesis [232].

All examples of genes and gene combinations that have been discussed above are by no means an exhaustive list of potentially effective and safe targets for therapeutic overexpression. Seeming contradictions in the effects resulting from the overexpression of some antioxidant genes on stress resistance and carcinogenesis were, apparently, caused by a number of non-obvious factors. In some cases, different effects of overexpression of the same gene could have risen from functional differences between the isoforms of product protein created by alternative splicing of the same gene. For example, the activity of invasion and metastasis of colon cancer cells increased when a splice variant b of the *GLRX3* gene (Txl-2b) was expressed, decreased when *GLRX3c* was expressed, and did not change during *GLRX3a* expression [294]. The activities of different isoforms of glutaminase, which is involved in the glutathione-dependent antioxidant defense system of the cell, could either promote tumor progression or suppress it through certain pathways involving miRNAs [347]. Besides different isoforms of proteins transcribed from one gene, non-coding circular RNAs (circRNAs) that have regulatory functions can be transcribed. For example, circRNA transcribed from the *SOD2* gene plays an important role in hepatocellular carcinoma progression [348]. Therefore, different functional properties of different transcripts of the gene must be taken into account when choosing cDNA for the overexpression of the gene by a classic method via the introduction of an additional copy of the gene or when designing guide RNAs for promoters of genes that have several alternative transcription initiation sites (such as *SOD2*) for CRISPRa technology application. However, these features of genes are discussed far less than the effects of the simultaneous overexpression of functional gene combinations are.

Nevertheless, the selection of only one splice variant of the gene is not supposed to be a perfect decision. Dysregulation of the gene’s expression because of the introduction of cDNA under a synthetic promoter or another gene’s promoter can cause contradicting effects, as, obviously, the degree of overexpression, its changes with time, and the presence or absence of a transcriptional response to various signals would affect the phenotypic manifestation of the gene. Preserving the chromosome context and, to some degree, the natural regulation when overexpressing a cell’s own genes with technologies, such as CRISPRa, could provide better results. In addition to that, CRISPRa allows the simultaneous overexpression of combinations of a number of genes. Presently, there are very few studies in which the researchers have overexpressed a cells’ own antioxidant genes.

Even the stage of tumor development may determine contradictions in the effects of antioxidant defense gene overexpression, apart from those related to the functional properties of different transcripts of the same gene and such obvious factors as the genetic context and the tissue (if it is a local gene therapy). Thus, *SOD2* overexpression in mice with benign thyroid tumors resulted in an increased tumor burden. In contrast, in mice with aggressive follicular thyroid cancer, the overexpression of *SOD2* reduced tumor proliferation and improved mortality rates [259].

Knowledge of the cytoprotective potential of even those genes whose overexpression has a pronounced pro-oncogenic effect can be of high applied value. For example, the overexpression of *PON2* in most experimental systems led to cancer promotion [313,314,315,316]. The overexpression of *PON2* is found in many tumors and often correlates with tumor aggressiveness and poor prognosis [315,316,349]. At the same time, a unique set of properties of the product of this gene, including the ability to activate glucose transport [314,316] and to suppress oxidative stress and apoptosis [350], makes its temporary or local activation a promising manipulation to reduce the damage caused by ischemia-reperfusion [143].

## 6. Conclusions

The complexity and ambiguity of the effects of antioxidant defense gene overexpression also resulted from the multifunctionality of product proteins. In addition to their main function of ROS and organic radical detoxification, they are frequent participants in signal cascades and are regulators of other protein activities [90,104,194], including those that occur due to changes in ROS levels as signaling molecules [1,2,6]. Thus, the overexpression of individual genes and their combinations that encode the effectors of the antioxidant system seems to be more promising than attempts to regulate the transcription factors affecting them, such as *NFE2L2*. Furthermore, common approaches based on weak effects on multiple functionally complementary targets, for example, when using plant extracts [351,352,353] in the therapy of various diseases, seem to be more practically applicable than those with strong effects on a single target.

## Figures and Tables

**Table 1:** Influence of antioxidant gene overexpression on resistance to oxidative and genotoxic stress factors in vitro and in vivo.

Gene	Model	Effect on Resistance
*ALDH3A1*	in vitro	Increased resistance: 4-hydroxyperoxycyclophosphamide, doxorubicin, etoposide, 5-FU, γ-ray, H_2_O_2_ [72]
*CAT*	in vitro	Increased resistance: UVB [73], benzo(a)pyrene [74], paraquat [81], H_2_O_2_ [83,89,90,91], benzyl isothiocyanate [84], dexamethasone [86], temozolomide, γ-ray, staurosporine [88], combination of ascorbate and menadione [90] Unchanged resistance: γ-ray [82], paraquat, TBHP [83], TNF-alpha [85], γ-ray, 5-FU, cisplatin, doxorubicin [90] Decreased resistance: Cr (VI) [87], paclitaxel, etoposide, arsenic trioxide, aminotriazol [90]
	in vivo	Increased resistance: benzo(a)pyrene [75,76], H_2_O_2_ [77], doxorubicin [78], proton radiation [79,80] Unchanged resistance: γ-ray [82]
*SOD1 +* *CAT*	in vivo	Increased resistance: benzo(a)pyren [75]
*GCLC*	in vitro	Increased resistance: γ-ray [93], UVC [94], tamoxifen [95]
	in vivo	Increased resistance: paraquat [92]
*GCLC+* *GCLM*	in vitro	Increased resistance: γ-ray [93]
*GCLM*	in vitro	Increased resistance: γ-ray [93]
*GPX1*	in vitro	Increased resistance: UVB [96], deoxynivalenol [97], cisplatin [99,103,105], X-ray [100], doxorubicin [106], H_2_O_2_, GSH high concentration [107] Decreased resistance: cisplatin [103], gemcitabine [104]
	in vivo	Increased resistance: ischemia-reperfusion injury [98], cocaine [101], microcystin-leucine-arginine [102]
*GPX2*	in vitro	Increased resistance: cisplatin [108]
*GPX3*	in vitro	Increased resistance: ascorbate [109], cisplatin [110], NAPQI [111]
*GPX4*	in vitro	Increased resistance: hypericin + PDT [112], TBHP [113], H_2_O_2_, erastin [114], AAPH, 5-HPETE [115], staurosporine, etoposide, UVB, actinomycin D, cycloheximide [116] Unchanged resistance: etoposide [113], calcium ionophore A23187 [116]
*GSR*	in vitro	Increased resistance: temozolomide, cisplatin [117], H_2_O_2_ [118]
*GSTP1*	in vitro	Increased resistance: hypericin+PDT [112]
*mt-PRDX5*	in vitro	Increased resistance: H_2_O_2_ [119]
*mt-SOD1*	in vitro	Increased resistance: γ-ray [51]
*mt-CAT*	in vitro	Increased resistance: γ-ray [82]
*mt-CAT + SOD2*	in vitro	Increased resistance: γ-ray [82]
*MTI*	in vitro	Increased resistance: TBHP, CdCl_2_ [120], Zn(II) [121] Unchanged resistance: alkylating agents [121]
*MTII*	in vitro	Increased resistance: MNU, MNNG [122], cisplatin, melphalan, chlorambucil [123], streptozotocin [124] Unchanged resistance: bleomycin [122,123], γ-ray, MMS, N-hydroxyethyl N-chloroethylnitrosourea [122], 5-FU, vincristine, doxorubicin [123]
*NFE2L2*	in vitro	Increased resistance: UVA+UVB [125], cisplatin [126,128,129,130], 5-FU [126,130], γ-ray [126], TBHP [127], X-ray [73], tamoxifen [131], gemcitabine [132]
*NQO1*	in vitro	Increased resistance: tamoxifen [95]
*nuclear-PRDX5*	in vitro	Unchanged resistance: H_2_O_2_ [119]
PON1	in vitro	Increased resistance: 5-fluorouracil, paclitaxel, cisplatin, etoposide [133], streptozotocin (mouse model of diabetes) [138]
	in vivo	Increased resistance: arthritis induction (K/BxN serum transfer (STIA) or collagen antibody transfer (CAIA) to mice [134], carbon tetrachloride [135,137], elastase-induced abdominal aortic aneurysm [136]
PON2	in vitro	Increased resistance: y-ray [139], tert-butyl-hydroperoxide [140], cisplatin [141], dexamethasone [142], oxygen-glucose deprivation/reoxygenation exposure [143], Nε-(carboxymethyl) lysine [144], pyocyanin [145], hypoxia [146], 2,3-dimethoxy-1,4-naphthoquinone [148] Unchanged resistance: dacarbazine [141]
	in vivo	Increased resistance: dexamethasone [142], apolipoprotein E deficiency [147]
PON3	in vivo	Increased resistance: carbon tetrachloride [135,149]
*PRDX1*	in vitro	Increased resistance: thiosemicarbazone iron chelator triapine [150], glucose oxidase [151], BCNU [62], γ-ray [65,152], 6-hydroxydopamine [152], doxorubicin [154]
*PRDX2*	in vitro	Increased resistance: H_2_O_2_ [58,155], γ-ray [156], 6-hydroxydopamine [153], doxorubicin [154]
	in vivo	Increased resistance: 6-hydroxydopamine [153], ischemia [176]
*PRDX3*	in vitro	Increased resistance: H_2_O_2_ [157], doxorubicin [154], arsenic trioxide [158] Unchanged resistance: 6-hydroxydopamine [153]
*PRDX4*	in vitro	Increased resistance: γ-ray [66], urethane [159], photon radiation [160], 6-hydroxydopamine [153] Unchanged resistance: doxorubicin [154]
*PRDX5*	in vitro	Increased resistance: H_2_O_2_ [161,162,165], TBHP [162], MPP+ [163], adriamycin, etoposide [164], menadione, cigarette smoke extract [165], shikonin [166], emodin [167], β-Lapachone [168], doxorubicin [154]
*PRDX6*	in vitro	Increased resistance: cisplatin [169], doxorubicin [170], hypoxia, cobalt chloride [171], UVA, menadione [172], H_2_O_2_ [173], X-ray [175] Decreased resistance: TNF-α/CHX solution [173]
	in vivo	Increased resistance: UVA, UVB [172], urethane [174]
*SLC31A1*	in vitro	Increased resistance: paraquat [177]
*SLC7A11*	in vitro	Increased resistance: cisplatin [178], temozolomide [179]
*SOD1*	in vitro	Increased resistance: γ-ray [181,183], S-nitroso-N-acetylpenicillamine, spermine-NONOate, diethylamine-NONOate [182], paraquat [83,177], cisplatin [186,187], docosahexaenoic acid [188], xanthine oxidase, hypoxanthine, menadione [189], TBHP [83], benzyl isothiocyanate [84], TNF and hyperthermia [190], Bortezomib [191], phenazine methosulfate [192] Unchanged resistance: γ-ray [51,180,193], N-methyl-D-aspartate, kainic acid, glutamate [182], H_2_O_2_ [83,182], UVB [73] Decreased resistance: menadione [182], Cr (VI) [87]
	in vivo	Increased resistance: paraquat, γ-ray [184], benzo(a)pyrene [75,76] Unchanged resistance: hyperoxia, paraquat [185]
*SOD1 + CAT*	in vitro	Increased resistance: paraquat, H_2_O_2_ [83], UVB [73] Unchanged resistance: TBHP [83]
*SOD2*	in vitro	Increased resistance: paraquat [180], γ-ray [51,82,181,195,196,203,205,206], cisplatin [194,201], X-ray [197], doxorubicin [198,200], docetaxel [200], 2-methoxyestradiol [202], glutamate [56], TNF- alpha [85], H_2_O_2_ [204], menadione, epirubicin [207] Unchanged resistance: γ-ray [193,206] Decreased resistance: Cr (VI) [87]
	in vivo	Increased resistance: γ-ray [51,82] Unchanged resistance: 100% O_2_ [199]
mt-signal deleted *SOD2*	in vitro	Unchanged resistance: γ-ray [51]
*SOD2 + CAT*	in vitro	Unchanged resistance: TNF-alpha [85]
*SOD3*	in vitro	Increased resistance: H_2_O_2_ [211]
	in vivo	Increased resistance: 4-MV photon radiation [208], ischemia [209], 12-O-tetradecanoylphorbol-13-acetate [210], lipopolysaccharide induced endotoxemia [20]
*TXN*	in vitro	Increased resistance: H_2_O_2_ [212,220,224], daunomycin [213], MPP+ [215], menadione, 1-chloro-2,4-dinitrobenzene [71], arsenic trioxide [222,223] Unchanged resistance: dexamethasone, doxorubicin, etoposide [220], auranofin, juglone [71]
	in vivo	Increased resistance: methamphetamine [214,221], sepsis-induced myocardial dysfunction, sham surgery [216], ischemia [217,225], ex vivo ischemia [218], adriamycin [219]
*TXNL1*	in vitro	Decreased resistance: cisplatin [226]
*TXNRD1*	in vitro	Increased resistance: X-ray [227], H_2_O_2_ [224]

## Supplementary Materials

The following supporting information can be downloaded at: https://www.mdpi.com/article/10.3390/antiox11122316/s1.

## References

[1] Cui Q., Wang J.-Q., Assaraf Y.G., Ren L., Gupta P., Wei L., Ashby C.R., Yang D.-H., Chen Z.-S. (2018). Modulating ROS to Overcome Multidrug Resistance in Cancer. Drug Resist. Update.

[2] Kasai S., Shimizu S., Tatara Y., Mimura J., Itoh K. (2020). Regulation of Nrf2 by Mitochondrial Reactive Oxygen Species in Physiology and Pathology. Biomolecules.

[3] Banoth B., Cassel S.L. (2018). Mitochondria in Innate Immune Signaling. Transl. Res..

[4] Poljsak B., Šuput D., Milisav I. (2013). Achieving the Balance between ROS and Antioxidants: When to Use the Synthetic Antioxidants. Oxidative Med. Cell. Longev..

[5] Kudryavtseva A.V., Krasnov G.S., Dmitriev A.A., Alekseev B.Y., Kardymon O.L., Sadritdinova A.F., Fedorova M.S., Pokrovsky A.V., Melnikova N.V., Kaprin A.D. (2016). Mitochondrial Dysfunction and Oxidative Stress in Aging and Cancer. Oncotarget.

[6] Księżakowska-Łakoma K., Żyła M., Wilczyński J.R. (2014). Mitochondrial Dysfunction in Cancer. Menopausal Rev..

[7] Chaiswing L., St. Clair W.H., St. Clair D.K. (2018). Redox Paradox: A Novel Approach to Therapeutics-Resistant Cancer. Antioxid. Redox Signal..

[8] Quagliariello V., Coppola C., Mita D.G., Piscopo G., Iaffaioli R.V., Botti G., Maurea N. (2019). Low Doses of Bisphenol A Have Pro-Inflammatory and pro-Oxidant Effects, Stimulate Lipid Peroxidation and Increase the Cardiotoxicity of Doxorubicin in Cardiomyoblasts. Environ. Toxicol. Pharmacol..

[9] Reuter S., Gupta S.C., Chaturvedi M.M., Aggarwal B.B. (2010). Oxidative Stress, Inflammation, and Cancer: How Are They Linked?. Free. Radic. Biol. Med..

[10] Vaziri N.D. (2008). Causal Link between Oxidative Stress, Inflammation, and Hypertension. Iran. J. Kidney Dis..

[11] Federico A., Morgillo F., Tuccillo C., Ciardiello F., Loguercio C. (2007). Chronic Inflammation and Oxidative Stress in Human Carcinogenesis. Int. J. Cancer.

[12] Poprac P., Jomova K., Simunkova M., Kollar V., Rhodes C.J., Valko M. (2017). Targeting Free Radicals in Oxidative Stress-Related Human Diseases. Trends Pharmacol. Sci..

[13] Marczin N., El-Habashi N., Hoare G.S., Bundy R.E., Yacoub M. (2003). Antioxidants in Myocardial Ischemia–reperfusion Injury: Therapeutic Potential and Basic Mechanisms. Arch. Biochem. Biophys..

[14] Chen Z., Zhong C. (2014). Oxidative Stress in Alzheimer’s Disease. Neurosci. Bull..

[15] Dhapola R., Sarma P., Medhi B., Prakash A., Reddy D.H. (2022). Recent Advances in Molecular Pathways and Therapeutic Implications Targeting Mitochondrial Dysfunction for Alzheimer’s Disease. Mol. Neurobiol..

[16] Yang N., Guan Q.-W., Chen F.-H., Xia Q.-X., Yin X.-X., Zhou H.-H., Mao X.-Y. (2020). Antioxidants Targeting Mitochondrial Oxidative Stress: Promising Neuroprotectants for Epilepsy. Oxidative Med. Cell. Longev..

[17] Zheng J., Winderickx J., Franssens V., Liu B. (2018). A Mitochondria-Associated Oxidative Stress Perspective on Huntington’s Disease. Front. Mol. Neurosci..

[18] Tang Q., Liu H., Shi X.-J., Cheng Y. (2020). Blood Oxidative Stress Marker Aberrations in Patients with Huntington’s Disease: A Meta-Analysis Study. Oxidative Med. Cell. Longev..

[19] Asmat U., Abad K., Ismail K. (2016). Diabetes Mellitus and Oxidative Stress—A Concise Review. Saudi Pharm. J..

[20] Call J.A., Donet J., Martin K.S., Sharma A.K., Chen X., Zhang J., Cai J., Galarreta C.A., Okutsu M., Du Z. (2017). Muscle-Derived Extracellular Superoxide Dismutase Inhibits Endothelial Activation and Protects against Multiple Organ Dysfunction Syndrome in Mice. Free Radic. Biol. Med..

[21] Xu F., Xu J., Xiong X., Deng Y. (2019). Salidroside Inhibits MAPK, NF-ΚB, and STAT3 Pathways in Psoriasis-Associated Oxidative Stress via SIRT1 Activation. Redox Rep..

[22] Kharaeva Z., Gostova E., De Luca C., Raskovic D., Korkina L. (2009). Clinical and Biochemical Effects of Coenzyme Q10, Vitamin E, and Selenium Supplementation to Psoriasis Patients. Nutrition.

[23] Baek J.-O., Byamba D., Wu W.H., Kim T.-G., Lee M.-G. (2012). Assessment of an Imiquimod-Induced Psoriatic Mouse Model in Relation to Oxidative Stress. Arch. Dermatol. Res..

[24] Gabr S.A., Al-Ghadir A.H. (2012). Role of Cellular Oxidative Stress and Cytochrome c in the Pathogenesis of Psoriasis. Arch. Dermatol. Res..

[25] Baral H., Sekiguchi A., Uchiyama A., Nisaa Amalia S., Yamazaki S., Inoue Y., Yokoyama Y., Ogino S., Torii R., Hosoi M. (2021). Inhibition of Skin Fibrosis in Systemic Sclerosis by Botulinum Toxin B via the Suppression of Oxidative Stress. J. Dermatol..

[26] Finkel T., Holbrook N.J. (2000). Oxidants, Oxidative Stress and the Biology of Ageing. Nature.

[27] Schriner S.E., Linford N.J. (2006). Extension of Mouse Lifespan by Overexpression of Catalase. Age.

[28] Poljsak B. (2011). Strategies for Reducing or Preventing the Generation of Oxidative Stress. Oxidative Med. Cell. Longev..

[29] García-Sánchez A., Miranda-Díaz A.G., Cardona-Muñoz E.G. (2020). The Role of Oxidative Stress in Physiopathology and Pharmacological Treatment with Pro- and Antioxidant Properties in Chronic Diseases. Oxidative Med. Cell. Longev..

[30] Hamilton C.A., Miller W.H., Al-Benna S., Julia BROSNAN M., Drummond R.D., McBRIDE M.W., Dominiczak A.F. (2004). Strategies to Reduce Oxidative Stress in Cardiovascular Disease. Clin. Sci..

[31] Münzel T., Daiber A. (2018). Environmental Stressors and Their Impact on Health and Disease with Focus on Oxidative Stress. Antioxid. Redox Signal..

[32] Gupta S.C., Patchva S., Aggarwal B.B. (2013). Therapeutic Roles of Curcumin: Lessons Learned from Clinical Trials. AAPS J..

[33] Yucel C., Quagliariello V., Iaffaioli R.V., Ferrari G., Donsì F. (2015). Submicron Complex Lipid Carriers for Curcumin Delivery to Intestinal Epithelial Cells: Effect of Different Emulsifiers on Bioaccessibility and Cell Uptake. Int. J. Pharm..

[34] Elejalde E., Villarán M.C., Alonso R.M. (2021). Grape Polyphenols Supplementation for Exercise-Induced Oxidative Stress. J. Int. Soc. Sport. Nutr..

[35] Joseph S.V., Edirisinghe I., Burton-Freeman B.M. (2016). Fruit Polyphenols: A Review of Anti-Inflammatory Effects in Humans. Crit. Rev. Food Sci. Nutr..

[36] Li G., Ding K., Qiao Y., Zhang L., Zheng L., Pan T., Zhang L. (2020). Flavonoids Regulate Inflammation and Oxidative Stress in Cancer. Molecules.

[37] Efferth T., Oesch F. (2022). Anti-Inflammatory and Anti-Cancer Activities of Frankincense: Targets, Treatments and Toxicities. Semin. Cancer Biol..

[38] Gutierrez-Mariscal F.M., Arenas-de Larriva A.P., Limia-Perez L., Romero-Cabrera J.L., Yubero-Serrano E.M., López-Miranda J. (2020). Coenzyme Q10 Supplementation for the Reduction of Oxidative Stress: Clinical Implications in the Treatment of Chronic Diseases. Int. J. Mol. Sci..

[39] Anand David A., Arulmoli R., Parasuraman S. (2016). Overviews of Biological Importance of Quercetin: A Bioactive Flavonoid. Phcog. Rev..

[40] Tardiolo G., Bramanti P., Mazzon E. (2018). Overview on the Effects of N-Acetylcysteine in Neurodegenerative Diseases. Molecules.

[41] Costa N.T., Iriyoda T.M.V., Alfieri D.F., Simão A.N.C., Dichi I. (2018). Influence of Disease-Modifying Antirheumatic Drugs on Oxidative and Nitrosative Stress in Patients with Rheumatoid Arthritis. Inflammopharmacology.

[42] Patterson C., Madamanchi N.R., Runge M.S. (2000). The Oxidative Paradox: Another Piece in the Puzzle. Circ. Res..

[43] Li N., Wu X., Zhuang W., Xia L., Chen Y., Wu C., Rao Z., Du L., Zhao R., Yi M. (2021). Tomato and Lycopene and Multiple Health Outcomes: Umbrella Review. Food Chem..

[44] Suhett L.G., de Miranda Monteiro Santos R., Silveira B.K.S., Leal A.C.G., de Brito A.D.M., de Novaes J.F., Lucia C.M.D. (2021). Effects of Curcumin Supplementation on Sport and Physical Exercise: A Systematic Review. Crit. Rev. Food Sci. Nutr..

[45] Parks N.E., Jackson-Tarlton C.S., Vacchi L., Merdad R., Johnston B.C. (2020). Dietary Interventions for Multiple Sclerosis-Related Outcomes. Cochrane Database Syst. Rev..

[46] Ramdas W.D. (2018). The Relation between Dietary Intake and Glaucoma: A Systematic Review. Acta Ophthalmol..

[47] Jenkins D.J.A., Kitts D., Giovannucci E.L., Sahye-Pudaruth S., Paquette M., Blanco Mejia S., Patel D., Kavanagh M., Tsirakis T., Kendall C.W.C. (2020). Selenium, Antioxidants, Cardiovascular Disease, and All-Cause Mortality: A Systematic Review and Meta-Analysis of Randomized Controlled Trials. Am. J. Clin. Nutr..

[48] Jenkins D.J.A., Spence J.D., Giovannucci E.L., Kim Y., Josse R., Vieth R., Blanco Mejia S., Viguiliouk E., Nishi S., Sahye-Pudaruth S. (2018). Supplemental Vitamins and Minerals for CVD Prevention and Treatment. J. Am. Coll. Cardiol..

[49] Yang J., Fernández-Galilea M., Martínez-Fernández L., González-Muniesa P., Pérez-Chávez A., Martínez J.A., Moreno-Aliaga M.J. (2019). Oxidative Stress and Non-Alcoholic Fatty Liver Disease: Effects of Omega-3 Fatty Acid Supplementation. Nutrients.

[50] Aune D., Keum N., Giovannucci E., Fadnes L.T., Boffetta P., Greenwood D.C., Tonstad S., Vatten L.J., Riboli E., Norat T. (2018). Dietary Intake and Blood Concentrations of Antioxidants and the Risk of Cardiovascular Disease, Total Cancer, and All-Cause Mortality: A Systematic Review and Dose-Response Meta-Analysis of Prospective Studies. Am. J. Clin. Nutr..

[51] Greenberger J.S., Epperly M.W., Gretton J., Jefferson M., Nie S., Bernarding M., Kagan V., Guo H.L. (2003). Radioprotective Gene Therapy. Curr. Gene Ther..

[52] Velegzhaninov I., Ievlev V., Pylina Y., Shadrin D., Vakhrusheva O. (2018). Programming of Cell Resistance to Genotoxic and Oxidative Stress. Biomedicines.

[53] Schäfer M., Farwanah H., Willrodt A., Huebner A.J., Sandhoff K., Roop D., Hohl D., Bloch W., Werner S. (2012). Nrf2 Links Epidermal Barrier Function with Antioxidant Defense. EMBO Mol. Med..

[54] Usui S., Oveson B.C., Iwase T., Lu L., Lee S.Y., Jo Y.-J., Wu Z., Choi E.-Y., Samulski R.J., Campochiaro P.A. (2011). Overexpression of SOD in Retina: Need for Increase in H_2_O_2_-Detoxifying Enzyme in Same Cellular Compartment. Free Radic. Biol. Med..

[55] Snezhkina A.V., Kudryavtseva A.V., Kardymon O.L., Savvateeva M.V., Melnikova N.V., Krasnov G.S., Dmitriev A.A. (2019). ROS Generation and Antioxidant Defense Systems in Normal and Malignant Cells. Oxidative Med. Cell. Longev..

[56] Fukui M., Zhu B.T. (2010). Mitochondrial Superoxide Dismutase SOD2, but Not Cytosolic SOD1, Plays a Critical Role in Protection against Glutamate-Induced Oxidative Stress and Cell Death in HT22 Neuronal Cells. Free Radic. Biol. Med..

[57] Sharma V., Joseph C., Ghosh S., Agarwal A., Mishra M.K., Sen E. (2007). Kaempferol Induces Apoptosis in Glioblastoma Cells through Oxidative Stress. Mol. Cancer Ther..

[58] Lu W., Fu Z., Wang H., Feng J., Wei J., Guo J. (2014). Peroxiredoxin 2 Is Upregulated in Colorectal Cancer and Contributes to Colorectal Cancer Cells’ Survival by Protecting Cells from Oxidative Stress. Mol. Cell Biochem..

[59] Neumann C.A., Krause D.S., Carman C.V., Das S., Dubey D.P., Abraham J.L., Bronson R.T., Fujiwara Y., Orkin S.H., Van Etten R.A. (2003). Essential Role for the Peroxiredoxin Prdx1 in Erythrocyte Antioxidant Defence and Tumour Suppression. Nature.

[60] Song L.-L., Tu Y.-Y., Xia L., Wang W.-W., Wei W., Ma C.-M., Wen D.-H., Lei H., Xu H.-Z., Wu Y.-L. (2014). Targeting Catalase but Not Peroxiredoxins Enhances Arsenic Trioxide-Induced Apoptosis in K562 Cells. PLoS ONE.

[61] Smith-Pearson P.S., Kooshki M., Spitz D.R., Poole L.B., Zhao W., Robbins M.E. (2008). Decreasing Peroxiredoxin II Expression Decreases Glutathione, Alters Cell Cycle Distribution, and Sensitizes Glioma Cells to Ionizing Radiation and H_2_O_2_. Free Radic. Biol. Med..

[62] Poschmann G., Grzendowski M., Stefanski A., Bruns E., Meyer H.E., Stühler K. (2015). Redox Proteomics Reveal Stress Responsive Proteins Linking Peroxiredoxin-1 Status in Glioma to Chemosensitivity and Oxidative Stress. Biochim. Biophys. Acta (BBA) Proteins Proteom..

[63] Duan J., Lang Y., Song C., Xiong J., Wang Y., Yan Y. (2013). SiRNA Targeting of PRDX3 Enhances Cisplatin-Induced Apoptosis in Ovarian Cancer Cells through the Suppression of the NF-ΚB Signaling Pathway. Mol. Med. Rep..

[64] Cerda M.B., Lloyd R., Batalla M., Giannoni F., Casal M., Policastro L. (2017). Silencing Peroxiredoxin-2 Sensitizes Human Colorectal Cancer Cells to Ionizing Radiation and Oxaliplatin. Cancer Lett..

[65] Chen M.-F., Keng P.C., Shau H., Wu C.-T., Hu Y.-C., Liao S.-K., Chen W.-C. (2006). Inhibition of Lung Tumor Growth and Augmentation of Radiosensitivity by Decreasing Peroxiredoxin I Expression. Int. J. Radiat. Oncol. Biol. Phys..

[66] Kim T.H., Song J., Alcantara Llaguno S.R., Murnan E., Liyanarachchi S., Palanichamy K., Yi J.-Y., Viapiano M.S., Nakano I., Yoon S.O. (2012). Suppression of Peroxiredoxin 4 in Glioblastoma Cells Increases Apoptosis and Reduces Tumor Growth. PLoS ONE.

[67] Gao M., Jia X., Wu Q., Cheng Y., Chen F., Zhang J. (2011). Silencing Prx1 and/or Prx5 Sensitizes Human Esophageal Cancer Cells to Ionizing Radiation and Increases Apoptosis via Intracellular ROS Accumulation. Acta Pharm. Sin..

[68] Xiang L., Xie G., Liu C., Zhou J., Chen J., Yu S., Li J., Pang X., Shi H., Liang H. (2013). Knock-down of Glutaminase 2 Expression Decreases Glutathione, NADH, and Sensitizes Cervical Cancer to Ionizing Radiation. Biochim. Biophys. Acta (BBA) Mol. Cell Res..

[69] Priestley J.R.C., Kautenburg K.E., Casati M.C., Endres B.T., Geurts A.M., Lombard J.H. (2016). The NRF2 Knockout Rat: A New Animal Model to Study Endothelial Dysfunction, Oxidant Stress, and Microvascular Rarefaction. Am. J. Physiol. Heart Circ. Physiol..

[70] Schwartz P.J., Reaume A., Scott R., Coyle J.T. (1998). Effects of Over- and under-Expression of Cu,Zn-Superoxide Dismutase on the Toxicity of Glutamate Analogs in Transgenic Mouse Striatum. Brain Res..

[71] Eriksson S.E., Prast-Nielsen S., Flaberg E., Szekely L., Arnér E.S.J. (2009). High Levels of Thioredoxin Reductase 1 Modulate Drug-Specific Cytotoxic Efficacy. Free Radic. Biol. Med..

[72] Voulgaridou G.-P., Kiziridou M., Mantso T., Chlichlia K., Galanis A., Koukourakis M.I., Franco R., Panayiotidis M.I., Pappa A. (2016). Aldehyde Dehydrogenase 3A1 Promotes Multi-Modality Resistance and Alters Gene Expression Profile in Human Breast Adenocarcinoma MCF-7 Cells. Int. J. Biochem. Cell Biol..

[73] Rezvani H.R., Mazurier F., Cario-André M., Pain C., Ged C., Taïeb A., de Verneuil H. (2006). Protective Effects of Catalase Overexpression on UVB-Induced Apoptosis in Normal Human Keratinocytes. J. Biol. Chem..

[74] Yang F., Yang H., Ramesh A., Goodwin J.S., Okoro E.U., Guo Z. (2016). Overexpression of Catalase Enhances Benzo(a)Pyrene Detoxification in Endothelial Microsomes. PLoS ONE.

[75] Yang H., Zhou L., Wang Z., Roberts L.J., Lin X., Zhao Y., Guo Z. (2009). Overexpression of Antioxidant Enzymes in ApoE-Deficient Mice Suppresses Benzo(a)Pyrene-Accelerated Atherosclerosis. Atherosclerosis.

[76] Wang Z., Yang H., Ramesh A., Roberts L.J., Zhou L., Lin X., Zhao Y., Guo Z. (2009). Overexpression of Cu/Zn-Superoxide Dismutase and/or Catalase Accelerates Benzo(a)Pyrene Detoxification by Upregulation of the Aryl Hydrocarbon Receptor in Mouse Endothelial Cells. Free Radic. Biol. Med..

[77] Sun J., Tower J. (1999). FLP Recombinase-Mediated Induction of Cu/Zn-Superoxide Dismutase Transgene Expression Can Extend the Life Span of Adult Drosophila Melanogaster Flies. Mol. Cell Biol..

[78] Kang Y.J., Chen Y., Epstein P.N. (1996). Suppression of Doxorubicin Cardiotoxicity by Overexpression of Catalase in the Heart of Transgenic Mice. J. Biol. Chem..

[79] Liao A.C., Craver B.M., Tseng B.P., Tran K.K., Parihar V.K., Acharya M.M., Limoli C.L. (2013). Mitochondrial-Targeted Human Catalase Affords Neuroprotection from Proton Irradiation. Radiat. Res..

[80] Parihar V.K., Allen B.D., Tran K.K., Chmielewski N.N., Craver B.M., Martirosian V., Morganti J.M., Rosi S., Vlkolinsky R., Acharya M.M. (2015). Targeted Overexpression of Mitochondrial Catalase Prevents Radiation-Induced Cognitive Dysfunction. Antioxid. Redox Signal..

[81] Narasimhan M., Riar A.K., Rathinam M.L., Vedpathak D., Henderson G., Mahimainathan L. (2014). Hydrogen Peroxide Responsive MiR153 Targets Nrf2/ARE Cytoprotection in Paraquat Induced Dopaminergic Neurotoxicity. Toxicol. Lett..

[82] Epperly M.W., Melendez J.A., Zhang X., Nie S., Pearce L., Peterson J., Franicola D., Dixon T., Greenberger B.A., Komanduri P. (2009). Mitochondrial Targeting of a Catalase Transgene Product by Plasmid Liposomes Increases Radioresistance In Vitro and In Vivo. Radiat. Res..

[83] Mele J., Van Remmen H., Vijg J., Richardson A. (2006). Characterization of Transgenic Mice That Overexpress Both Copper Zinc Superoxide Dismutase and Catalase. Antioxid. Redox Signal..

[84] Xiao D., Powolny A.A., Singh S.V. (2008). Benzyl Isothiocyanate Targets Mitochondrial Respiratory Chain to Trigger Reactive Oxygen Species-Dependent Apoptosis in Human Breast Cancer Cells. J. Biol. Chem..

[85] Dasgupta J., Subbaram S., Connor K.M., Rodriguez A.M., Tirosh O., Beckman J.S., Jourd’Heuil D., Melendez J.A. (2006). Manganese Superoxide Dismutase Protects from TNF-Alpha-Induced Apoptosis by Increasing the Steady-State Production of H_2_O_2_. Antioxid. Redox Signal..

[86] Tome M.E., Baker A.F., Powis G., Payne C.M., Briehl M.M. (2001). Catalase-Overexpressing Thymocytes Are Resistant to Glucocorticoid-Induced Apoptosis and Exhibit Increased Net Tumor Growth1. Cancer Res..

[87] Son Y.-O., Pratheeshkumar P., Wang L., Wang X., Fan J., Kim D.-H., Lee J.-Y., Zhang Z., Lee J.-C., Shi X. (2013). Reactive Oxygen Species Mediate Cr(VI)-Induced Carcinogenesis through PI3K/AKT-Dependent Activation of GSK-3β/β-Catenin Signaling. Toxicol. Appl. Pharm..

[88] Flor S., Oliva C.R., Ali M.Y., Coleman K.L., Greenlee J.D., Jones K.A., Monga V., Griguer C.E. (2021). Catalase Overexpression Drives an Aggressive Phenotype in Glioblastoma. Antioxidants.

[89] Finch J.S., Tome M.E., Kwei K.A., Bowden G.T. (2006). Catalase Reverses Tumorigenicity in a Malignant Cell Line by an Epidermal Growth Factor Receptor Pathway. Free Radic. Biol. Med..

[90] Glorieux C., Dejeans N., Sid B., Beck R., Calderon P.B., Verrax J. (2011). Catalase Overexpression in Mammary Cancer Cells Leads to a Less Aggressive Phenotype and an Altered Response to Chemotherapy. Biochem. Pharmacol..

[91] Herpers B.L., Schrama L.H., Kaal E.C., Joosten E.A., Dop Bär P.R. (1999). Microinjection of Catalase CDNA Prevents Hydrogen Peroxide-Induced Motoneuron Death. Neuroreport.

[92] Moskalev A., Shaposhnikov M., Proshkina E., Belyi A., Fedintsev A., Zhikrivetskaya S., Guvatova Z., Sadritdinova A., Snezhkina A., Krasnov G. (2016). The Influence of Pro-Longevity Gene Gclc Overexpression on the Age-Dependent Changes in Drosophila Transcriptome and Biological Functions. BMC Genom..

[93] Cortes-Wanstreet M.M., Giedzinski E., Limoli C.L., Luderer U. (2009). Overexpression of Glutamate–cysteine Ligase Protects Human COV434 Granulosa Tumour Cells against Oxidative and γ-Radiation-Induced Cell Death. Mutagenesis.

[94] Fan Y., Wu D., Jin L., Yin Z. (2005). Human Glutamylcysteine Synthetase Protects HEK293 Cells against UV-Induced Cell Death through Inhibition of c-Jun NH2-Terminal Kinase. Cell Biol. Int..

[95] Fiorillo M., Sotgia F., Sisci D., Cappello A.R., Lisanti M.P. (2017). Mitochondrial “Power” Drives Tamoxifen Resistance: NQO1 and GCLC Are New Therapeutic Targets in Breast Cancer. Oncotarget.

[96] Baliga M.S., Wang H., Zhuo P., Schwartz J.L., Diamond A.M. (2007). Selenium and GPx-1 Overexpression Protect Mammalian Cells against UV-Induced DNA Damage. Biol. Trace Elem. Res..

[97] Ren Z., Chen C., Fan Y., Chen C., He H., Wang X., Zhang Z., Zuo Z., Peng G., Hu Y. (2019). Toxicity of DON on GPx1-Overexpressed or Knockdown Porcine Splenic Lymphocytes In Vitro and Protective Effects of Sodium Selenite. Oxidative Med. Cell. Longev..

[98] Weisbrot-Lefkowitz M., Reuhl K., Perry B., Chan P.H., Inouye M., Mirochnitchenko O. (1998). Overexpression of Human Glutathione Peroxidase Protects Transgenic Mice against Focal Cerebral Ischemia/Reperfusion Damage. Mol. Brain Res..

[99] Chen B., Shen Z., Wu D., Xie X., Xu X., Lv L., Dai H., Chen J., Gan X. (2019). Glutathione Peroxidase 1 Promotes NSCLC Resistance to Cisplatin via ROS-Induced Activation of PI3K/AKT Pathway. BioMed Res. Int..

[100] Yang W., Shen Y., Wei J., Liu F. (2015). MicroRNA-153/Nrf-2/GPx1 Pathway Regulates Radiosensitivity and Stemness of Glioma Stem Cells via Reactive Oxygen Species. Oncotarget.

[101] Mai H.N., Chung Y.H., Shin E.-J., Kim D.-J., Sharma N., Lee Y.J., Jeong J.H., Nah S.-Y., Jang C.-G., Kim H.-C. (2019). Glutathione Peroxidase-1 Overexpressing Transgenic Mice Are Protected from Cocaine-Induced Drug Dependence. Neurochem. Int..

[102] Shin E.-J., Hwang Y.G., Pham D.T., Lee J.W., Lee Y.J., Pyo D., Jeong J.H., Lei X.G., Kim H.-C. (2018). Glutathione Peroxidase-1 Overexpressing Transgenic Mice Are Protected from Neurotoxicity Induced by Microcystin-Leucine-Arginine. Environ. Toxicol..

[103] Gan X., Chen B., Shen Z., Liu Y., Li H., Xie X., Xu X., Li H., Huang Z., Chen J. (2014). High GPX1 Expression Promotes Esophageal Squamous Cell Carcinoma Invasion, Migration, Proliferation and Cisplatin-Resistance but Can Be Reduced by Vitamin D. Int. J. Clin. Exp. Med..

[104] Meng Q., Shi S., Liang C., Liang D., Hua J., Zhang B., Xu J., Yu X. (2018). Abrogation of Glutathione Peroxidase-1 Drives EMT and Chemoresistance in Pancreatic Cancer by Activating ROS-Mediated Akt/GSK3β/Snail Signaling. Oncogene.

[105] Huang Z., Liu Y., Huang Z., Li H., Gan X., Shen Z. (2016). 1,25-Dihydroxyvitamin D3 Alleviates Salivary Adenoid Cystic Carcinoma Progression by Suppressing GPX1 Expression through the NF-ΚB Pathway. Int. J. Oncol..

[106] Gouazé V., Mirault M.E., Carpentier S., Salvayre R., Levade T., Andrieu-Abadie N. (2001). Glutathione Peroxidase-1 Overexpression Prevents Ceramide Production and Partially Inhibits Apoptosis in Doxorubicin-Treated Human Breast Carcinoma Cells. Mol. Pharm..

[107] Faucher K., Rabinovitch-Chable H., Barrière G., Cook-Moreau J., Rigaud M. (2003). Overexpression of Cytosolic Glutathione Peroxidase (GPX1) Delays Endothelial Cell Growth and Increases Resistance to Toxic Challenges. Biochimie.

[108] Du H., Chen B., Jiao N.-L., Liu Y.-H., Sun S.-Y., Zhang Y.-W. (2020). Elevated Glutathione Peroxidase 2 Expression Promotes Cisplatin Resistance in Lung Adenocarcinoma. Oxidative Med. Cell. Longev..

[109] Worley B.L., Kim Y.S., Mardini J., Zaman R., Leon K.E., Vallur P.G., Nduwumwami A., Warrick J.I., Timmins P.F., Kesterson J.P. (2018). GPx3 Supports Ovarian Cancer Progression by Manipulating the Extracellular Redox Environment. Redox Biol..

[110] Saga Y., Ohwada M., Suzuki M., Konno R., Kigawa J., Ueno S., Mano H. (2008). Glutathione Peroxidase 3 Is a Candidate Mechanism of Anticancer Drug Resistance of Ovarian Clear Cell Adenocarcinoma. Oncol. Rep..

[111] Kanno S.-I., Tomizawa A., Yomogida S., Hara A. (2017). Glutathione Peroxidase 3 Is a Protective Factor against Acetaminophen-Induced Hepatotoxicity in Vivo and in Vitro. Int. J. Mol. Med..

[112] Theodossiou T.A., Olsen C.E., Jonsson M., Kubin A., Hothersall J.S., Berg K. (2017). The Diverse Roles of Glutathione-Associated Cell Resistance against Hypericin Photodynamic Therapy. Redox Biol..

[113] Kinowaki Y., Kurata M., Ishibashi S., Ikeda M., Tatsuzawa A., Yamamoto M., Miura O., Kitagawa M., Yamamoto K. (2018). Glutathione Peroxidase 4 Overexpression Inhibits ROS-Induced Cell Death in Diffuse Large B-Cell Lymphoma. Lab. Investig..

[114] Peng G., Tang Z., Xiang Y., Chen W. (2019). Glutathione Peroxidase 4 Maintains a Stemness Phenotype, Oxidative Homeostasis and Regulates Biological Processes in Panc-1 Cancer Stem-like Cells. Oncol. Rep..

[115] Imai H., Sumi D., Sakamoto H., Hanamoto A., Arai M., Chiba N., Nakagawa Y. (1996). Overexpression of Phospholipid Hydroperoxide Glutathione Peroxidase Suppressed Cell Death Due to Oxidative Damage in Rat Basophile Leukemia Cells (RBL-2H3). Biochem. Biophys. Res. Commun..

[116] Nomura K., Imai H., Koumura T., Arai M., Nakagawa Y. (1999). Mitochondrial Phospholipid Hydroperoxide Glutathione Peroxidase Suppresses Apoptosis Mediated by a Mitochondrial Death Pathway. J. Biol. Chem..

[117] Zhu Z., Du S., Du Y., Ren J., Ying G., Yan Z. (2018). Glutathione Reductase Mediates Drug Resistance in Glioblastoma Cells by Regulating Redox Homeostasis. J. Neurochem..

[118] Kim S.-J., Jung H.-J., Hyun D.-H., Park E.-H., Kim Y.-M., Lim C.-J. (2010). Glutathione Reductase Plays an Anti-Apoptotic Role against Oxidative Stress in Human Hepatoma Cells. Biochimie.

[119] Banmeyer I., Marchand C., Clippe A., Knoops B. (2005). Human Mitochondrial Peroxiredoxin 5 Protects from Mitochondrial DNA Damages Induced by Hydrogen Peroxide. FEBS Lett..

[120] Schwarz M.A., Lazo J.S., Yalowich J.C., Reynolds I., Kagan V.E., Tyurin V., Kim Y.M., Watkins S.C., Pitt B.R. (1994). Cytoplasmic Metallothionein Overexpression Protects NIH 3T3 Cells from Tert-Butyl Hydroperoxide Toxicity. J. Biol. Chem..

[121] Goncharova E.I., Rossman T.G. (1994). A Role for Metallothionein and Zinc in Spontaneous Mutagenesis. Cancer Res..

[122] Kaina B., Lohrer H., Karin M., Herrlich P. (1990). Overexpressed Human Metallothionein IIA Gene Protects Chinese Hamster Ovary Cells from Killing by Alkylating Agents. Proc. Natl. Acad. Sci. USA.

[123] Kelley S.L., Basu A., Teicher B.A., Hacker M.P., Hamer D.H., Lazo J.S. (1988). Overexpression of Metallothionein Confers Resistance to Anticancer Drugs. Science.

[124] Chen H., Carlson E.C., Pellet L., Moritz J.T., Epstein P.N. (2001). Overexpression of Metallothionein in Pancreatic Beta-Cells Reduces Streptozotocin-Induced DNA Damage and Diabetes. Diabetes.

[125] Xian D., Xiong X., Xu J., Xian L., Lei Q., Song J., Zhong J. (2019). Nrf2 Overexpression for the Protective Effect of Skin-Derived Precursors against UV-Induced Damage: Evidence from a Three-Dimensional Skin Model. Oxidative Med. Cell. Longev..

[126] Lister A., Nedjadi T., Kitteringham N.R., Campbell F., Costello E., Lloyd B., Copple I.M., Williams S., Owen A., Neoptolemos J.P. (2011). Nrf2 Is Overexpressed in Pancreatic Cancer: Implications for Cell Proliferation and Therapy. Mol. Cancer.

[127] Zou X., Feng Z., Li Y., Wang Y., Wertz K., Weber P., Fu Y., Liu J. (2012). Stimulation of GSH Synthesis to Prevent Oxidative Stress-Induced Apoptosis by Hydroxytyrosol in Human Retinal Pigment Epithelial Cells: Activation of Nrf2 and JNK-P62/SQSTM1 Pathways. J. Nutr. Biochem..

[128] Syu J.-P., Chi J.-T., Kung H.-N. (2016). Nrf2 Is the Key to Chemotherapy Resistance in MCF7 Breast Cancer Cells under Hypoxia. Oncotarget.

[129] Cai L., Jin X., Zhang J., Li L., Zhao J. (2020). Metformin Suppresses Nrf2-Mediated Chemoresistance in Hepatocellular Carcinoma Cells by Increasing Glycolysis. Aging.

[130] Samatiwat P., Prawan A., Senggunprai L., Kukongviriyapan U., Kukongviriyapan V. (2016). Nrf2 Inhibition Sensitizes Cholangiocarcinoma Cells to Cytotoxic and Antiproliferative Activities of Chemotherapeutic Agents. Tumor Biol..

[131] Kim S.K., Yang J.W., Kim M.R., Roh S.H., Kim H.G., Lee K.Y., Jeong H.G., Kang K.W. (2008). Increased Expression of Nrf2/ARE-Dependent Anti-Oxidant Proteins in Tamoxifen-Resistant Breast Cancer Cells. Free Radic. Biol. Med..

[132] Ju H.-Q., Gocho T., Aguilar M., Wu M., Zhuang Z.-N., Fu J., Yanaga K., Huang P., Chiao P.J. (2015). Mechanisms of Overcoming Intrinsic Resistance to Gemcitabine in Pancreatic Ductal Adenocarcinoma through the Redox Modulation. Mol. Cancer Ther..

[133] Aldonza M.B.D., Son Y.S., Sung H.-J., Ahn J.M., Choi Y.-J., Kim Y.-I., Cho S., Cho J.-Y. (2017). Paraoxonase-1 (PON1) Induces Metastatic Potential and Apoptosis Escape via Its Antioxidative Function in Lung Cancer Cells. Oncotarget.

[134] Charles-Schoeman C., Wang J., Shahbazian A., Lee Y.Y., Wang X., Grijalva V., Brahn E., Shih D.M., Devarajan A., Montano C. (2020). Suppression of Inflammatory Arthritis in Human Serum Paraoxonase 1 Transgenic Mice. Sci. Rep..

[135] Peng W., Zhang C., Lv H., Zhu J., Zang Y., Pang X., Zhang J., Qin J. (2010). Comparative Evaluation of the Protective Potentials of Human Paraoxonase 1 and 3 against CCl4-Induced Liver Injury. Toxicol. Lett..

[136] Burillo E., Tarin C., Torres-Fonseca M.-M., Fernandez-García C.-E., Martinez-Pinna R., Martinez-Lopez D., Llamas-Granda P., Camafeita E., Lopez J.A., Vega de Ceniga M. (2016). Paraoxonase-1 Overexpression Prevents Experimental Abdominal Aortic Aneurysm Progression. Clin. Sci..

[137] Zhang C., Peng W., Jiang X., Chen B., Zhu J., Zang Y., Zhang J., Zhu T., Qin J. (2008). Transgene Expression of Human PON1 Q in Mice Protected the Liver against CCl4-Induced Injury. J. Gene Med..

[138] Rozenberg O., Shiner M., Aviram M., Hayek T. (2008). Paraoxonase 1 (PON1) Attenuates Diabetes Development in Mice through Its Antioxidative Properties. Free Radic. Biol. Med..

[139] Krüger M., Pabst A.M., Al-Nawas B., Horke S., Moergel M. (2015). Paraoxonase-2 (PON2) Protects Oral Squamous Cell Cancer Cells against Irradiation-Induced Apoptosis. J. Cancer Res. Clin. Oncol..

[140] Bacchetti T., Sartini D., Pozzi V., Cacciamani T., Ferretti G., Emanuelli M. (2017). Exploring the Role of Paraoxonase-2 in Bladder Cancer: Analyses Performed on Tissue Samples, Urines and Cell Culturess. Oncotarget.

[141] Campagna R., Bacchetti T., Salvolini E., Pozzi V., Molinelli E., Brisigotti V., Sartini D., Campanati A., Ferretti G., Offidani A. (2020). Paraoxonase-2 Silencing Enhances Sensitivity of A375 Melanoma Cells to Treatment with Cisplatin. Antioxidants.

[142] Hui P.-Y., Chen Y.-H., Qin J., Jiang X.-H. (2022). PON2 Blockade Overcomes Dexamethasone Resistance in Acute Lymphoblastic Leukemia. Hematology.

[143] Bai J., Jia P., Zhang Y., Wang K., Wu G. (2021). Paraoxonase 2 Protects against Oxygen-Glucose Deprivation/Reoxygenation-Induced Neuronal Injury by Enhancing Nrf2 Activation via GSK-3β Modulation. Hum. Exp. Toxicol..

[144] Ravi R., Subramaniam Rajesh B. (2022). Paraoxonase 2 Protects against the CML Mediated Mitochondrial Dysfunction through Modulating JNK Pathway in Human Retinal Cells. Biochim. Biophys. Acta (BBA) Gen. Subj..

[145] Horke S., Witte I., Altenhöfer S., Wilgenbus P., Goldeck M., Förstermann U., Xiao J., Kramer G.L., Haines D.C., Chowdhary P.K. (2010). Paraoxonase 2 Is Down-Regulated by the *Pseudomonas aeruginosa* Quorumsensing Signal *N*-(3-Oxododecanoyl)-L-Homoserine Lactone and Attenuates Oxidative Stress Induced by Pyocyanin. Biochem. J..

[146] Sulaiman D., Li J., Devarajan A., Cunningham C.M., Li M., Fishbein G.A., Fogelman A.M., Eghbali M., Reddy S.T. (2019). Paraoxonase 2 Protects against Acute Myocardial Ischemia-Reperfusion Injury by Modulating Mitochondrial Function and Oxidative Stress via the PI3K/Akt/GSK-3β RISK Pathway. J. Mol. Cell. Cardiol..

[147] Ng C.J., Hama S.Y., Bourquard N., Navab M., Reddy S.T. (2006). Adenovirus Mediated Expression of Human Paraoxonase 2 Protects against the Development of Atherosclerosis in Apolipoprotein E-Deficient Mice. Mol. Genet. Metab..

[148] Horke S., Witte I., Wilgenbus P., Krüger M., Strand D., Förstermann U. (2007). Paraoxonase-2 Reduces Oxidative Stress in Vascular Cells and Decreases Endoplasmic Reticulum Stress–Induced Caspase Activation. Circulation.

[149] Peng W., Jiang X., Haiqin L., Zhang C., Zhu J., Zhang J., Zang Y., Qin J. (2009). Protective Effects of Transgene Expressed Human PON3 against CCl4-Induced Subacute Liver Injury in Mice. Biomed. Pharmacother..

[150] Myers C.R. (2016). Enhanced Targeting of Mitochondrial Peroxide Defense by the Combined Use of Thiosemicarbazones and Inhibitors of Thioredoxin Reductase. Free Radic. Biol. Med..

[151] Klopotowska M., Bajor M., Graczyk-Jarzynka A., Kraft A., Pilch Z., Zhylko A., Firczuk M., Baranowska I., Lazniewski M., Plewczynski D. (2022). PRDX-1 Supports the Survival and Antitumor Activity of Primary and CAR-Modified NK Cells under Oxidative Stress. Cancer Immunol. Res.

[152] Kim Y.-J., Lee W.-S., Ip C., Chae H.-Z., Park E.-M., Park Y.-M. (2006). Prx1 Suppresses Radiation-Induced c-Jun NH2-Terminal Kinase Signaling in Lung Cancer Cells through Interaction with the Glutathione S-Transferase Pi/c-Jun NH2-Terminal Kinase Complex. Cancer Res..

[153] Hu X., Weng Z., Chu C.T., Zhang L., Cao G., Gao Y., Signore A., Zhu J., Hastings T., Greenamyre J.T. (2011). Peroxiredoxin-2 Protects against 6-Hydroxydopamine-Induced Dopaminergic Neurodegeneration via Attenuation of the Apoptosis Signal-Regulating Kinase (ASK1) Signaling Cascade. J. Neurosci..

[154] McDonald C., Muhlbauer J., Perlmutter G., Taparra K., Phelan S.A. (2014). Peroxiredoxin Proteins Protect MCF-7 Breast Cancer Cells from Doxorubicin-Induced Toxicity. Int. J. Oncol..

[155] Gao T., Che X.-X., Wang R., Xiao C.-S., Jia Y.-P. (2020). Protective Effect of Overexpression of PrxII on H_2_O_2_-Induced Cardiomyocyte Injury. Eur. Rev. Med. Pharm. Sci..

[156] Wang T., Tamae D., LeBon T., Shively J.E., Yen Y., Li J.J. (2005). The Role of Peroxiredoxin II in Radiation-Resistant MCF-7 Breast Cancer Cells. Cancer Res..

[157] Wang Y.-G., Li L., Liu C.-H., Hong S., Zhang M.-J. (2014). Peroxiredoxin 3 Is Resistant to Oxidation-Induced Apoptosis of Hep-3b Cells. Clin. Transl. Oncol..

[158] Vivas-Mejía P.E., Ozpolat B., Chen X., Lopez-Berestein G. (2009). Downregulation of the C-MYC Target Gene, Peroxiredoxin III, Contributes to Arsenic Trioxide-Induced Apoptosis in Acute Promyelocytic Leukemia. Int. J. Cancer.

[159] Zheng J., Guo X., Nakamura Y., Zhou X., Yamaguchi R., Zhang J., Ishigaki Y., Uramoto H., Yamada S. (2020). Overexpression of PRDX4 Modulates Tumor Microenvironment and Promotes Urethane-Induced Lung Tumorigenesis. Oxidative Med. Cell. Longev..

[160] Park J.J., Chang H.W., Jeong E.-J., Roh J.-L., Choi S.-H., Jeon S.-Y., Ko G.H., Kim S.Y. (2009). Peroxiredoxin IV Protects Cells from Radiation-Induced Apoptosis in Head-and-Neck Squamous Cell Carcinoma. Int. J. Radiat. Oncol. Biol. Phys..

[161] Yuan J., Murrell G.A.C., Trickett A., Landtmeters M., Knoops B., Wang M.-X. (2004). Overexpression of Antioxidant Enzyme Peroxiredoxin 5 Protects Human Tendon Cells against Apoptosis and Loss of Cellular Function during Oxidative Stress. Biochim. Biophys. Acta.

[162] Banmeyer I., Marchand C., Verhaeghe C., Vucic B., Rees J.-F., Knoops B. (2004). Overexpression of Human Peroxiredoxin 5 in Subcellular Compartments of Chinese Hamster Ovary Cells: Effects on Cytotoxicity and DNA Damage Caused by Peroxides. Free Radic. Biol. Med..

[163] De Simoni S., Linard D., Hermans E., Knoops B., Goemaere J. (2013). Mitochondrial Peroxiredoxin-5 as Potential Modulator of Mitochondria-ER Crosstalk in MPP^+^-Induced Cell Death. J. Neurochem..

[164] Kropotov A., Gogvadze V., Shupliakov O., Tomilin N., Serikov V.B., Tomilin N.V., Zhivotovsky B. (2006). Peroxiredoxin V Is Essential for Protection against Apoptosis in Human Lung Carcinoma Cells. Exp. Cell Res..

[165] Avila P.C., Kropotov A.V., Krutilina R., Krasnodembskay A., Tomilin N.V., Serikov V.B. (2008). Peroxiredoxin V Contributes to Antioxidant Defense of Lung Epithelial Cells. Lung.

[166] Chandimali N., Sun H.-N., Kong L.-Z., Zhen X., Liu R., Kwon T., Lee D.-S. (2019). Shikonin-Induced Apoptosis of Colon Cancer Cells Is Reduced by Peroxiredoxin V Expression. Anticancer Res..

[167] Jin Y.-Z., Sun H.-N., Liu Y., Lee D.-H., Kim J.-S., Kim S.-U., Jiao B.-Y., Han Y.-H., Jin M.-H., Shen G.-N. (2019). Peroxiredoxin V Inhibits Emodin-Induced Gastric Cancer Cell Apoptosis via the ROS/Bcl2 Pathway. In Vivo.

[168] Liu Y., Kwon T., Kim J.-S., Chandimali N., Jin Y.-H., Gong Y.-X., Xie D.-P., Han Y.-H., Jin M.-H., Shen G.-N. (2019). Peroxiredoxin V Reduces β-Lapachone-Induced Apoptosis of Colon Cancer Cells. Anticancer Res..

[169] Pak J.H., Choi W.H., Lee H.M., Joo W.-D., Kim J.-H., Kim Y.-T., Kim Y.-M., Nam J.-H. (2011). Peroxiredoxin 6 Overexpression Attenuates Cisplatin-Induced Apoptosis in Human Ovarian Cancer Cells. Cancer Investig..

[170] Fang Z., Liu T., Liu X., Lu Y., Sun Y., Xiao R., Fan R., Liu L. (2019). PRDX6 Promotes Proliferation and Induces Chemo-Resistance via Peroxidase Activity in Toledo Diffuse Large B-Cell Lymphoma Cells. Transl. Cancer Res..

[171] Tulsawani R., Kelly L.S., Fatma N., Chhunchha B., Kubo E., Kumar A., Singh D.P. (2010). Neuroprotective Effect of Peroxiredoxin 6 against Hypoxia-Induced Retinal Ganglion Cell Damage. BMC Neurosci..

[172] Kümin A., Huber C., Rülicke T., Wolf E., Werner S. (2006). Peroxiredoxin 6 Is a Potent Cytoprotective Enzyme in the Epidermis. Am. J. Pathol..

[173] Xu X., Lu D., Zhuang R., Wei X., Xie H., Wang C., Zhu Y., Wang J., Zhong C., Zhang X. (2016). The Phospholipase A2 Activity of Peroxiredoxin 6 Promotes Cancer Cell Death Induced by Tumor Necrosis Factor Alpha in Hepatocellular Carcinoma. Mol. Carcinog..

[174] Yun H.-M., Park K.-R., Park M.H., Kim D.H., Jo M.R., Kim J.Y., Kim E.-C., Yoon D.Y., Han S.B., Hong J.T. (2015). PRDX6 Promotes Tumor Development via the JAK2/STAT3 Pathway in a Urethane-Induced Lung Tumor Model. Free Radic. Biol. Med..

[175] He Y., Xu W., Xiao Y., Pan L., Chen G., Tang Y., Zhou J., Wu J., Zhu W., Zhang S. (2018). Overexpression of Peroxiredoxin 6 (PRDX6) Promotes the Aggressive Phenotypes of Esophageal Squamous Cell Carcinoma. J. Cancer.

[176] Gan Y., Ji X., Hu X., Luo Y., Zhang L., Li P., Liu X., Yan F., Vosler P., Gao Y. (2012). Transgenic Overexpression of Peroxiredoxin-2 Attenuates Ischemic Neuronal Injury via Suppression of a Redox-Sensitive pro-Death Signaling Pathway. Antioxid. Redox Signal..

[177] Reczek C.R., Birsoy K., Kong H., Martínez-Reyes I., Wang T., Gao P., Sabatini D.M., Chandel N.S. (2017). A CRISPR Screen Identifies a Pathway Required for Paraquat-Induced Cell Death. Nat. Chem. Biol..

[178] Zhang P., Wang W., Wei Z., Xu L., Yang X., Du Y. (2016). XCT Expression Modulates Cisplatin Resistance in Tca8113 Tongue Carcinoma Cells. Oncol. Lett..

[179] Polewski M.D., Reveron-Thornton R.F., Cherryholmes G.A., Marinov G.K., Aboody K.S. (2017). SLC7A11 Overexpression in Glioblastoma Is Associated with Increased Cancer Stem Cell-Like Properties. Stem. Cells Dev..

[180] Veldwijk M.R., Trah J., Wang M., Maier P., Fruehauf S., Zeller W.J., Herskind C., Wenz F. (2011). Overexpression of Manganese Superoxide Dismutase Does Not Increase Clonogenic Cell Survival despite Effect on Apoptosis in Irradiated Lymphoblastoid Cells. Radiat. Res..

[181] Veldwijk M.R., Herskind C., Sellner L., Radujkovic A., Laufs S., Fruehauf S., Zeller W.J., Wenz F. (2009). Normal-Tissue Radioprotection by Overexpression of the Copper-Zinc and Manganese Superoxide Dismutase Genes. Strahlenther. Onkol..

[182] Ying W., Anderson C.M., Chen Y., Stein B.A., Fahlman C.S., Copin J.C., Chan P.H., Swanson R.A. (2000). Differing Effects of Copper, Zinc Superoxide Dismutase Overexpression on Neurotoxicity Elicited by Nitric Oxide, Reactive Oxygen Species, and Excitotoxins. J. Cereb. Blood Flow Metab..

[183] Gao Z., Sarsour E.H., Kalen A.L., Li L., Kumar M.G., Goswami P.C. (2008). Late ROS Accumulation and Radiosensitivity in SOD1-Overexpressing Human Glioma Cells. Free Radic. Biol. Med..

[184] Parkes T.L., Elia A.J., Dickinson D., Hilliker A.J., Phillips J.P., Boulianne G.L. (1998). Extension of Drosophila Lifespan by Overexpression of Human SOD1 in Motorneurons. Nat. Genet..

[185] Orr W.C., Sohal R.S. (1993). Effects of Cu-Zn Superoxide Dismutase Overexpression of Life Span and Resistance to Oxidative Stress in Transgenic Drosophila Melanogaster. Arch. Biochem. Biophys..

[186] Hour T.-C., Lai Y.-L., Kuan C.-I., Chou C.-K., Wang J.-M., Tu H.-Y., Hu H.-T., Lin C.-S., Wu W.-J., Pu Y.-S. (2010). Transcriptional Up-Regulation of SOD1 by CEBPD: A Potential Target for Cisplatin Resistant Human Urothelial Carcinoma Cells. Biochem. Pharm..

[187] Brown D.P.G., Chin-Sinex H., Nie B., Mendonca M.S., Wang M. (2009). Targeting Superoxide Dismutase 1 to Overcome Cisplatin Resistance in Human Ovarian Cancer. Cancer Chemother. Pharm..

[188] Ding W.-Q., Vaught J.L., Yamauchi H., Lind S.E. (2004). Differential Sensitivity of Cancer Cells to Docosahexaenoic Acid-Induced Cytotoxicity: The Potential Importance of down-Regulation of Superoxide Dismutase 1 Expression. Mol. Cancer Ther..

[189] Chen Y., Chan P.H., Swanson R.A. (2001). Astrocytes Overexpressing Cu,Zn Superoxide Dismutase Have Increased Resistance to Oxidative Injury. Glia.

[190] Li J.-J., Oberley L.W. (1997). Overexpression of Manganese-Containing Superoxide Dismutase Confers Resistance to the Cytotoxicity of Tumor Necrosis Factor α and/or Hyperthermia1. Cancer Res..

[191] Salem K., McCormick M.L., Wendlandt E., Zhan F., Goel A. (2014). Copper–zinc Superoxide Dismutase-Mediated Redox Regulation of Bortezomib Resistance in Multiple Myeloma. Redox Biol..

[192] Li S., Fu L., Tian T., Deng L., Li H., Xia W., Gong Q. (2018). Disrupting SOD1 Activity Inhibits Cell Growth and Enhances Lipid Accumulation in Nasopharyngeal Carcinoma. Cell Commun. Signal..

[193] Veldwijk M.R., Herskind C., Laufs S., Zeller W.J., Fruehauf S., Wenz F. (2004). Recombinant Adeno-Associated Virus 2-Mediated Transfer of the Human Superoxide-Dismutase Gene Does Not Confer Radioresistance on HeLa Cervical Carcinoma Cells. Radiother. Oncol..

[194] Chen P.-M., Cheng Y.-W., Wu T.-C., Chen C.-Y., Lee H. (2015). MnSOD Overexpression Confers Cisplatin Resistance in Lung Adenocarcinoma via the NF-ΚB/Snail/Bcl-2 Pathway. Free Radic. Biol. Med..

[195] Fisher C.J., Goswami P.C. (2008). Mitochondria-Targeted Antioxidant Enzyme Activity Regulates Radioresistance in Human Pancreatic Cancer Cells. Cancer Biol. Ther..

[196] Guo G., Yan-Sanders Y., Lyn-Cook B.D., Wang T., Tamae D., Ogi J., Khaletskiy A., Li Z., Weydert C., Longmate J.A. (2003). Manganese Superoxide Dismutase-Mediated Gene Expression in Radiation-Induced Adaptive Responses. Mol. Cell Biol..

[197] Motoori S., Majima H.J., Ebara M., Kato H., Hirai F., Kakinuma S., Yamaguchi C., Ozawa T., Nagano T., Tsujii H. (2001). Overexpression of Mitochondrial Manganese Superoxide Dismutase Protects against Radiation-Induced Cell Death in the Human Hepatocellular Carcinoma Cell Line HLE. Cancer Res..

[198] Hur G.-C., Cho S.J., Kim C.-H., Kim M.K., Bae S.I., Nam S.Y., Park J.-W., Kim W.H., Lee B.L. (2003). Manganese Superoxide Dismutase Expression Correlates with Chemosensitivity in Human Gastric Cancer Cell Lines. Clin. Cancer Res..

[199] Mockett R.J., Orr W.C., Rahmandar J.J., Benes J.J., Radyuk S.N., Klichko V.I., Sohal R.S. (1999). Overexpression of Mn-Containing Superoxide Dismutase in Transgenic Drosophila Melanogaster. Arch. Biochem. Biophys..

[200] Kumar A.P., Loo S.Y., Shin S.W., Tan T.Z., Eng C.B., Singh R., Putti T.C., Ong C.W., Salto-Tellez M., Goh B.C. (2014). Manganese Superoxide Dismutase Is a Promising Target for Enhancing Chemosensitivity of Basal-like Breast Carcinoma. Antioxid. Redox Signal..

[201] Zuo J., Zhao M., Liu B., Han X., Li Y., Wang W., Zhang Q., Lv P., Xing L., Shen H. (2019). TNF-α-mediated Upregulation of SOD-2 Contributes to Cell Proliferation and Cisplatin Resistance in Esophageal Squamous Cell Carcinoma. Oncol. Rep..

[202] Zhou J., Du Y. (2012). Acquisition of Resistance of Pancreatic Cancer Cells to 2-Methoxyestradiol Is Associated with the Upregulation of Manganese Superoxide Dismutase. Mol. Cancer Res..

[203] Hosoki A., Yonekura S.-I., Zhao Q.-L., Wei Z.-L., Takasaki I., Tabuchi Y., Wang L.-L., Hasuike S., Nomura T., Tachibana A. (2012). Mitochondria-Targeted Superoxide Dismutase (SOD2) Regulates Radiation Resistance and Radiation Stress Response in HeLa Cells. J. Radiat. Res..

[204] Reddy V.N., Kasahara E., Hiraoka M., Lin L.-R., Ho Y.-S. (2004). Effects of Variation in Superoxide Dismutases (SOD) on Oxidative Stress and Apoptosis in Lens Epithelium. Exp. Eye Res..

[205] Epperly M.W., Sikora C.A., DeFilippi S.J., Gretton J.A., Zhan Q., Kufe D.W., Greenberger J.S. (2002). Manganese Superoxide Dismutase (SOD2) Inhibits Radiation-Induced Apoptosis by Stabilization of the Mitochondrial Membrane. Radiat. Res..

[206] Zhang Z., Lang J., Cao Z., Li R., Wang X., Wang W. (2017). Radiation-Induced SOD2 Overexpression Sensitizes Colorectal Cancer to Radiation While Protecting Normal Tissue. Oncotarget.

[207] Kahlos K., Anttila S., Asikainen T., Kinnula K., Raivio K.O., Mattson K., Linnainmaa K., Kinnula V.L. (1998). Manganese Superoxide Dismutase in Healthy Human Pleural Mesothelium and in Malignant Pleural Mesothelioma. Am. J. Respir. Cell Mol. Biol..

[208] Rabbani Z.N., Anscher M.S., Folz R.J., Archer E., Huang H., Chen L., Golson M.L., Samulski T.S., Dewhirst M.W., Vujaskovic Z. (2005). Overexpression of Extracellular Superoxide Dismutase Reduces Acute Radiation Induced Lung Toxicity. BMC Cancer.

[209] Sheng H., Bart R., Oury T., Pearlstein R., Crapo J., Warner D. (1999). Mice Overexpressing Extracellular Superoxide Dismutase Have Increased Resistance to Focal Cerebral Ischemia. Neuroscience.

[210] Kim S.-H., Kim M.-O., Gao P., Youm C.A., Park H., Lee T.S., Kim K.-S., Suh J.-G., Lee H., Park B.-J. (2005). Overexpression of Extracellular Superoxide Dismutase (EC-SOD) in Mouse Skin Plays a Protective Role in DMBA/TPA-Induced Tumor Formation. Oncol. Res..

[211] Yang R., Wei L., Fu Q.-Q., Wang H., You H., Yu H.-R. (2016). SOD3 Ameliorates H_2_O_2_-Induced Oxidative Damage in SH-SY5Y Cells by Inhibiting the Mitochondrial Pathway. Neurochem. Res..

[212] Umeda-Kameyama Y., Tsuda M., Ohkura C., Matsuo T., Namba Y., Ohuchi Y., Aigaki T. (2007). Thioredoxin Suppresses Parkin-Associated Endothelin Receptor-like Receptor-Induced Neurotoxicity and Extends Longevity in Drosophila. J. Biol. Chem..

[213] Das K.C., Muniyappa H., Kundumani-Sridharan V., Subramani J. (2021). Thioredoxin Decreases Anthracycline Cardiotoxicity, But Sensitizes Cancer Cell Apoptosis. Cardiovasc. Toxicol..

[214] Yang L., Guo N., Fan W., Ni C., Huang M., Bai L., Zhang L., Zhang X., Wen Y., Li Y. (2020). Thioredoxin-1 Blocks Methamphetamine-Induced Injury in Brain through Inhibiting Endoplasmic Reticulum and Mitochondria-Mediated Apoptosis in Mice. NeuroToxicology.

[215] Bai L., Yan F., Deng R., Gu R., Zhang X., Bai J. (2021). Thioredoxin-1 Rescues MPP+/MPTP-Induced Ferroptosis by Increasing Glutathione Peroxidase 4. Mol. Neurobiol..

[216] Sánchez-Villamil J.P., D’Annunzio V., Finocchietto P., Holod S., Rebagliati I., Pérez H., Peralta J.G., Gelpi R.J., Poderoso J.J., Carreras M.C. (2016). Cardiac-Specific Overexpression of Thioredoxin 1 Attenuates Mitochondrial and Myocardial Dysfunction in Septic Mice. Int. J. Biochem. Cell Biol..

[217] Perez V., D’Annunzio V., Valdez L.B., Zaobornyj T., Bombicino S., Mazo T., Carbajosa N.L., Gironacci M.M., Boveris A., Sadoshima J. (2016). Thioredoxin-1 Attenuates Ventricular and Mitochondrial Postischemic Dysfunction in the Stunned Myocardium of Transgenic Mice. Antioxid. Redox Signal..

[218] D’Annunzio V., Perez V., Mazo T., Muñoz M.C., Dominici F.P., Carreras M.C., Poderoso J.J., Sadoshima J., Gelpi R.J. (2016). Loss of Myocardial Protection against Myocardial Infarction in Middle-Aged Transgenic Mice Overexpressing Cardiac Thioredoxin-1. Oncotarget.

[219] Shioji K., Kishimoto C., Nakamura H., Masutani H., Yuan Z., Oka S., Yodoi J. (2002). Overexpression of Thioredoxin-1 in Transgenic Mice Attenuates Adriamycin-Induced Cardiotoxicity. Circulation.

[220] Berggren M.I., Husbeck B., Samulitis B., Baker A.F., Gallegos A., Powis G. (2001). Thioredoxin Peroxidase-1 (Peroxiredoxin-1) Is Increased in Thioredoxin-1 Transfected Cells and Results in Enhanced Protection against Apoptosis Caused by Hydrogen Peroxide but Not by Other Agents Including Dexamethasone, Etoposide, and Doxorubicin. Arch. Biochem. Biophys..

[221] Huang M., Kong L., Yang L., Li X., Zhou X., Li Y., Bai J. (2018). The Role of Thioredoxin-1 in Resisting Methamphetamine-Induced Rewarding Effect. Behav. Brain Res..

[222] Park W. (2019). Upregulation of Thioredoxin and Its Reductase Attenuates Arsenic Trioxide-induced Growth Suppression in Human Pulmonary Artery Smooth Muscle Cells by Reducing Oxidative Stress. Oncol. Rep..

[223] Tian C., Gao P., Zheng Y., Yue W., Wang X., Jin H., Chen Q. (2008). Redox Status of Thioredoxin-1 (TRX1) Determines the Sensitivity of Human Liver Carcinoma Cells (HepG2) to Arsenic Trioxide-Induced Cell Death. Cell Res..

[224] Park W.H. (2019). Upregulated Thioredoxin and Its Reductase Prevent H_2_O_2_-Induced Growth Inhibition and Death in Human Pulmonary Artery Smooth Muscle Cells. Toxicol. In Vitro.

[225] Zhou F., Gomi M., Fujimoto M., Hayase M., Marumo T., Masutani H., Yodoi J., Hashimoto N., Nozaki K., Takagi Y. (2009). Attenuation of Neuronal Degeneration in Thioredoxin-1 Overexpressing Mice after Mild Focal Ischemia. Brain Res..

[226] Ni P., Xu W., Zhang Y., Chen Q., Li A., Wang S., Xu S., Zhou J. (2015). TXNL1 Induces Apoptosis in Cisplatin Resistant Human Gastric Cancer Cell Lines. Curr. Cancer Drug Targets.

[227] Zhang Y., Chen F., Tai G., Wang J., Shang J., Zhang B., Wang P., Huang B., Du J., Yu J. (2017). TIGAR Knockdown Radiosensitizes TrxR1-Overexpressing Glioma in Vitro and in Vivo via Inhibiting Trx1 Nuclear Transport. Sci. Rep..

[228] Cheng A.W., Wang H., Yang H., Shi L., Katz Y., Theunissen T.W., Rangarajan S., Shivalila C.S., Dadon D.B., Jaenisch R. (2013). Multiplexed Activation of Endogenous Genes by CRISPR-on, an RNA-Guided Transcriptional Activator System. Cell Res..

[229] Zhu F., Shao J., Tian Y., Xu Z. (2021). Sulfiredoxin-1 Protects Retinal Ganglion Cells from High Glucose-Induced Oxidative Stress and Inflammatory Injury by Potentiating Nrf2 Signaling via the Akt/GSK-3β Pathway. Int. Immunopharmacol..

[230] Rolfs F., Huber M., Gruber F., Böhm F., Pfister H.J., Bochkov V.N., Tschachler E., Dummer R., Hohl D., Schäfer M. (2013). Dual Role of the Antioxidant Enzyme Peroxiredoxin 6 in Skin Carcinogenesis. Cancer Res..

[231] Brigelius-Flohé R., Kipp A. (2009). Glutathione Peroxidases in Different Stages of Carcinogenesis. Biochim. Biophys. Acta (BBA) Gen. Subj..

[232] Castellone M.D., Langella A., Cantara S., Laurila J.P., Laatikainen L.E., Bellelli R., Pacini F., Salvatore M., Laukkanen M.O. (2014). Extracellular Superoxide Dismutase Induces Mouse Embryonic Fibroblast Proliferative Burst, Growth Arrest, Immortalization, and Consequent In Vivo Tumorigenesis. Antioxid. Redox Signal..

[233] Teoh M.L.T., Fitzgerald M.P., Oberley L.W., Domann F.E. (2009). Overexpression of Extracellular Superoxide Dismutase Attenuates Heparanase Expression and Inhibits Breast Carcinoma Cell Growth and Invasion. Cancer Res..

[234] Jeon Y.-J., Yoo H., Kim B.H., Lee Y.S., Jeon B., Kim S.-S., Kim T.-Y. (2012). IFNγ-Mediated Inhibition of Cell Proliferation through Increased PKCδ-Induced Overexpression of EC-SOD. BMB Rep..

[235] Li J., Liu Y., Liu Q. (2020). Expression of superoxide dismutase 2 in breast cancer and its clinical significance. Nan Fang Yi Ke Da Xue Xue Bao.

[236] Miar A., Hevia D., Muñoz-Cimadevilla H., Astudillo A., Velasco J., Sainz R.M., Mayo J.C. (2015). Manganese Superoxide Dismutase (SOD2/MnSOD)/Catalase and SOD2/GPx1 Ratios as Biomarkers for Tumor Progression and Metastasis in Prostate, Colon, and Lung Cancer. Free Radic. Biol. Med..

[237] Song Y.-H., Wang J., Chen Y.-J., Li X., Jiang X., Cao W.-H. (2017). MicroRNA-509-5p Functions as an Antioncogene in Breast Cancer via Targeting SOD2. Eur. Rev. Med. Pharm. Sci..

[238] Connor K.M., Hempel N., Nelson K.K., Dabiri G., Gamarra A., Belarmino J., Van De Water L., Mian B.M., Melendez J.A. (2007). Manganese Superoxide Dismutase Enhances the Invasive and Migratory Activity of Tumor Cells. Cancer Res..

[239] Kamarajugadda S., Cai Q., Chen H., Nayak S., Zhu J., He M., Jin Y., Zhang Y., Ai L., Martin S.S. (2013). Manganese Superoxide Dismutase Promotes Anoikis Resistance and Tumor Metastasis. Cell Death Dis..

[240] Li J.-J., Oberley L.W., St Clair D.K., Ridnour L.A., Oberley T.D. (1995). Phenotypic Changes Induced in Human Breast Cancer Cells by Overexpression of Manganese-Containing Superoxide Dismutase. Oncogene.

[241] Zhong W., Oberley L.W., Oberley T.D., Clair D.K.S. (1997). Suppression of the Malignant Phenotype of Human Glioma Cells by Overexpression of Manganese Superoxide Dismutase. Oncogene.

[242] Weydert C., Roling B., Liu J., Hinkhouse M.M., Ritchie J.M., Oberley L.W., Cullen J.J. (2003). Suppression of the Malignant Phenotype in Human Pancreatic Cancer Cells by the Overexpression of Manganese Superoxide Dismutase. Mol. Cancer Ther..

[243] Liu J., Hinkhouse M.M., Sun W., Weydert C.J., Ritchie J.M., Oberley L.W., Cullen J.J. (2004). Redox Regulation of Pancreatic Cancer Cell Growth: Role of Glutathione Peroxidase in the Suppression of the Malignant Phenotype. Hum. Gene Ther..

[244] Hu Y., Rosen D.G., Zhou Y., Feng L., Yang G., Liu J., Huang P. (2005). Mitochondrial Manganese-Superoxide Dismutase Expression in Ovarian Cancer. J. Biol. Chem..

[245] Hemachandra L.P.M.P., Shin D.-H., Dier U., Iuliano J.N., Engelberth S.A., Uusitalo L.M., Murphy S.K., Hempel N. (2015). Mitochondrial Superoxide Dismutase Has a Pro-Tumorigenic Role in Ovarian Clear Cell Carcinoma. Cancer Res..

[246] Nelson K.K., Ranganathan A.C., Mansouri J., Rodriguez A.M., Providence K.M., Rutter J.L., Pumiglia K., Bennett J.A., Melendez J.A. (2003). Elevated Sod2 Activity Augments Matrix Metalloproteinase Expression: Evidence for the Involvement of Endogenous Hydrogen Peroxide in Regulating Metastasis. Clin. Cancer Res..

[247] Li F., Wang H., Huang C., Lin J., Zhu G., Hu R., Feng H. (2011). Hydrogen Peroxide Contributes to the Manganese Superoxide Dismutase Promotion of Migration and Invasion in Glioma Cells. Free Radic. Res..

[248] Kattan Z., Minig V., Leroy P., Dauça M., Becuwe P. (2008). Role of Manganese Superoxide Dismutase on Growth and Invasive Properties of Human Estrogen-Independent Breast Cancer Cells. Breast Cancer Res. Treat..

[249] Lu Y.P., Lou Y.R., Yen P., Newmark H.L., Mirochnitchenko O.I., Inouye M., Huang M.T. (1997). Enhanced Skin Carcinogenesis in Transgenic Mice with High Expression of Glutathione Peroxidase or Both Glutathione Peroxidase and Superoxide Dismutase. Cancer Res..

[250] Liao X., Huang C., Zhang D., Wang J., Li J., Jin H., Huang C. (2017). Mitochondrial Catalase Induces Cells Transformation through Nucleolin-Dependent Cox-2 MRNA Stabilization. Free Radic. Biol. Med..

[251] Mishra M., Jiang H., Chawsheen H.A., Gerard M., Toledano M.B., Wei Q. (2018). Nrf2-Activated Expression of Sulfiredoxin Contributes to Urethane-Induced Lung Tumorigenesis. Cancer Lett..

[252] Gomez M.L., Shah N., Kenny T.C., Jenkins E.C., Germain D. (2019). SOD1 Is Essential for Oncogene-Driven Mammary Tumor Formation but Dispensable for Normal Development and Proliferation. Oncogene.

[253] Flores L.C., Roman M.G., Cunningham G.M., Cheng C., Dube S., Allen C., Van Remmen H., Hubbard G.B., Saunders T.L., Ikeno Y. (2018). Continuous Overexpression of Thioredoxin 1 Enhances Cancer Development and Does Not Extend Maximum Lifespan in Male C57BL/6 Mice. Pathobiol. Aging Age-Relat. Dis..

[254] Yi L., Shen H., Zhao M., Shao P., Liu C., Cui J., Wang J., Wang C., Guo N., Kang L. (2017). Inflammation-Mediated SOD-2 Upregulation Contributes to Epithelial-Mesenchymal Transition and Migration of Tumor Cells in Aflatoxin G1-Induced Lung Adenocarcinoma. Sci. Rep..

[255] Liu Z., Li S., Cai Y., Wang A., He Q., Zheng C., Zhao T., Ding X., Zhou X. (2012). Manganese Superoxide Dismutase Induces Migration and Invasion of Tongue Squamous Cell Carcinoma via H_2_O_2_-Dependent Snail Signaling. Free Radic. Biol. Med..

[256] Chang B., Yang H., Jiao Y., Wang K., Liu Z., Wu P., Li S., Wang A. (2016). SOD2 Deregulation Enhances Migration, Invasion and Has Poor Prognosis in Salivary Adenoid Cystic Carcinoma. Sci. Rep..

[257] Zhang Y., Zhang Y., Wang S., Li Q., Cao B., Huang B., Wang T., Guo R., Liu N. (2021). SP1-Induced LncRNA ZFPM2 Antisense RNA 1 (ZFPM2-AS1) Aggravates Glioma Progression via the MiR-515-5p/Superoxide Dismutase 2 (SOD2) Axis. Bioengineered.

[258] Dai J., Zhang S., Sun H., Wu Y., Yan M. (2021). LncRNA MAFG-AS1 Affects the Tumorigenesis of Breast Cancer Cells via the MiR-574-5p/SOD2 Axis. Biochem. Biophys. Res. Commun..

[259] Ashtekar A., Huk D., Magner A., La Perle K.M.D., Boucai L., Kirschner L.S. (2018). Alterations in Sod2-Induced Oxidative Stress Affect Endocrine Cancer Progression. J. Clin. Endocrinol. Metab..

[260] Fu A., Ma S., Wei N., Tan B.X.X., Tan E.Y., Luo K.Q. (2016). High Expression of MnSOD Promotes Survival of Circulating Breast Cancer Cells and Increases Their Resistance to Doxorubicin. Oncotarget.

[261] Jiang H., Wu L., Mishra M., Chawsheen H.A., Wei Q. (2014). Expression of Peroxiredoxin 1 and 4 Promotes Human Lung Cancer Malignancy. Am. J. Cancer Res..

[262] Niu W., Zhang M., Chen H., Wang C., Shi N., Jing X., Ge L., Chen T., Tang X. (2016). Peroxiredoxin 1 Promotes Invasion and Migration by Regulating Epithelial-to-Mesenchymal Transition during Oral Carcinogenesis. Oncotarget.

[263] Song C., Xiong G., Yang S., Wei X., Ye X., Huang W., Zhang R. (2020). PRDX1 Stimulates Non-Small-Cell Lung Carcinoma to Proliferate via the Wnt/β-Catenin Signaling. Panminerva Med..

[264] Gong F., Hou G., Liu H., Zhang M. (2015). Peroxiredoxin 1 Promotes Tumorigenesis through Regulating the Activity of MTOR/P70S6K Pathway in Esophageal Squamous Cell Carcinoma. Med. Oncol..

[265] Aguilar-Melero P., Prieto-Álamo M.-J., Jurado J., Holmgren A., Pueyo C. (2013). Proteomics in HepG2 Hepatocarcinoma Cells with Stably Silenced Expression of PRDX1. J. Proteom..

[266] Taniuchi K., Furihata M., Hanazaki K., Iwasaki S., Tanaka K., Shimizu T., Saito M., Saibara T. (2015). Peroxiredoxin 1 Promotes Pancreatic Cancer Cell Invasion by Modulating P38 MAPK Activity. Pancreas.

[267] Ha B., Kim E.-K., Kim J.-H., Lee H.N., Lee K.O., Lee S.Y., Jang H.H. (2012). Human Peroxiredoxin 1 Modulates TGF-Β1-Induced Epithelial–mesenchymal Transition through Its Peroxidase Activity. Biochem. Biophys. Res. Commun..

[268] Riddell J.R., Bshara W., Moser M.T., Spernyak J.A., Foster B.A., Gollnick S.O. (2011). Peroxiredoxin 1 Controls Prostate Cancer Growth through Toll-Like Receptor 4–Dependent Regulation of Tumor Vasculature. Cancer Res..

[269] Li H.-X., Sun X.-Y., Yang S.-M., Wang Q., Wang Z.-Y. (2018). Peroxiredoxin 1 Promoted Tumor Metastasis and Angiogenesis in Colorectal Cancer. Pathol. Res. Pract..

[270] Cai A.-L., Zeng W., Cai W.-L., Liu J.-L., Zheng X.-W., Liu Y., Yang X.-C., Long Y., Li J. (2018). Peroxiredoxin-1 Promotes Cell Proliferation and Metastasis through Enhancing Akt/MTOR in Human Osteosarcoma Cells. Oncotarget.

[271] Chen Q., Li J., Yang X., Ma J., Gong F., Liu Y. (2020). Prdx1 Promotes the Loss of Primary Cilia in Esophageal Squamous Cell Carcinoma. BMC Cancer.

[272] Song Y., Liu H., Cui C., Peng X., Wang C., Tian X., Li W. (2019). Silencing of Peroxiredoxin 1 Inhibits the Proliferation of Esophageal Cancer Cells and Promotes Apoptosis by Inhibiting the Activity of the PI3K/AKT Pathway. Cancer Manag. Res..

[273] Ho J.-N., Lee S.B., Lee S.-S., Yoon S.H., Kang G.Y., Hwang S.-G., Um H.-D. (2010). Phospholipase A2 Activity of Peroxiredoxin 6 Promotes Invasion and Metastasis of Lung Cancer Cells. Mol. Cancer Ther..

[274] Lee S.B., Ho J.-N., Yoon S.H., Kang G.Y., Hwang S.-G., Um H.-D. (2009). Peroxiredoxin 6 Promotes Lung Cancer Cell Invasion by Inducing Urokinase-Type Plasminogen Activator via P38 Kinase, Phosphoinositide 3-Kinase, and Akt. Mol. Cells.

[275] Schmitt A., Schmitz W., Hufnagel A., Schartl M., Meierjohann S. (2015). Peroxiredoxin 6 Triggers Melanoma Cell Growth by Increasing Arachidonic Acid-Dependent Lipid Signalling. Biochem. J..

[276] Chang X.-Z., Li D.-Q., Hou Y.-F., Wu J., Lu J.-S., Di G.-H., Jin W., Ou Z.-L., Shen Z.-Z., Shao Z.-M. (2007). Identification of the Functional Role of Peroxiredoxin 6 in the Progression of Breast Cancer. Breast Cancer Res..

[277] Yun H.-M., Park K.-R., Lee H.P., Lee D.H., Jo M., Shin D.H., Yoon D.-Y., Han S.B., Hong J.T. (2014). PRDX6 Promotes Lung Tumor Progression via Its GPx and IPLA2 Activities. Free Radic. Biol. Med..

[278] Mu R., Li Y., Xing J., Li Y., Lin R., Ye S., Zhang Y., Mu H., Guo X., An L. (2022). Effect of Lentivirus-Mediated Peroxiredoxins 6 Gene Silencing on the Phenotype of Human Gastric Cancer BGC-823 Cells. J. Cancer Res. Ther..

[279] Jing X., Du L., Niu A., Wang Y., Wang Y., Wang C. (2020). Silencing of PRDX2 Inhibits the Proliferation and Invasion of Non-Small Cell Lung Cancer Cells. BioMed Res. Int..

[280] Lu W., Fu Z., Wang H., Feng J., Wei J., Guo J. (2014). Peroxiredoxin 2 Knockdown by RNA Interference Inhibits the Growth of Colorectal Cancer Cells by Downregulating Wnt/β-Catenin Signaling. Cancer Lett..

[281] Chen Y., Yang S., Zhou H., Su D. (2020). PRDX2 Promotes the Proliferation and Metastasis of Non-Small Cell Lung Cancer In Vitro and In Vivo. BioMed Res. Int..

[282] Lv Z., Wei J., You W., Wang R., Shang J., Xiong Y., Yang H., Yang X., Fu Z. (2017). Disruption of the C-Myc/MiR-200b-3p/PRDX2 Regulatory Loop Enhances Tumor Metastasis and Chemotherapeutic Resistance in Colorectal Cancer. J. Transl. Med..

[283] Park Y.-H., Kim S.-U., Kwon T.-H., Kim J.-M., Song I.-S., Shin H.-J., Lee B.-K., Bang D.-H., Lee S.-J., Lee D.-S. (2016). Peroxiredoxin II Promotes Hepatic Tumorigenesis through Cooperation with Ras/Forkhead Box M1 Signaling Pathway. Oncogene.

[284] Kwon T., Kyung Rho J., Cheol Lee J., Park Y.-H., Shin H.-J., Cho S., Kang Y.-K., Kim B.-Y., Yoon D.-Y., Yu D.-Y. (2015). An Important Role for Peroxiredoxin II in Survival of A549 Lung Cancer Cells Resistant to Gefitinib. Exp. Mol. Med..

[285] Peng L., Xiong Y., Wang R., Xiang L., Zhou H., Fu Z. (2021). The Critical Role of Peroxiredoxin-2 in Colon Cancer Stem Cells. Aging.

[286] Wang W., Shen X.-B., Huang D.-B., Jia W., Liu W.-B., He Y.-F. (2019). Peroxiredoxin 4 Suppresses Anoikis and Augments Growth and Metastasis of Hepatocellular Carcinoma Cells through the β-Catenin/ID2 Pathway. Cell Oncol..

[287] Wei Q., Jiang H., Xiao Z., Baker A., Young M.R., Veenstra T.D., Colburn N.H. (2011). Sulfiredoxin-Peroxiredoxin IV Axis Promotes Human Lung Cancer Progression through Modulation of Specific Phosphokinase Signaling. Proc. Natl. Acad. Sci. USA.

[288] Chang K.-P., Yu J.-S., Chien K.-Y., Lee C.-W., Liang Y., Liao C.-T., Yen T.-C., Lee L.-Y., Huang L.-L., Liu S.-C. (2011). Identification of PRDX4 and P4HA2 as Metastasis-Associated Proteins in Oral Cavity Squamous Cell Carcinoma by Comparative Tissue Proteomics of Microdissected Specimens Using ITRAQ Technology. J. Proteome Res..

[289] Bhatia M., McGrath K.L., Di Trapani G., Charoentong P., Shah F., King M.M., Clarke F.M., Tonissen K.F. (2016). The Thioredoxin System in Breast Cancer Cell Invasion and Migration. Redox Biol..

[290] Raninga P.V., Di Trapani G., Vuckovic S., Bhatia M., Tonissen K.F. (2015). Inhibition of Thioredoxin 1 Leads to Apoptosis in Drug-Resistant Multiple Myeloma. Oncotarget.

[291] Cao M.-Q., You A.-B., Cui W., Zhang S., Guo Z.-G., Chen L., Zhu X.-D., Zhang W., Zhu X.-L., Guo H. (2020). Cross Talk between Oxidative Stress and Hypoxia via Thioredoxin and HIF-2α Drives Metastasis of Hepatocellular Carcinoma. FASEB J..

[292] Shang W., Xie Z., Lu F., Fang D., Tang T., Bi R., Chen L., Jiang L. (2019). Increased Thioredoxin-1 Expression Promotes Cancer Progression and Predicts Poor Prognosis in Patients with Gastric Cancer. Oxidative Med. Cell. Longev..

[293] He F., Wei L., Luo W., Liao Z., Li B., Zhou X., Xiao X., You J., Chen Y., Zheng S. (2016). Glutaredoxin 3 Promotes Nasopharyngeal Carcinoma Growth and Metastasis via EGFR/Akt Pathway and Independent of ROS. Oncotarget.

[294] Lu Y., Zhao X., Li K., Luo G., Nie Y., Shi Y., Zhou Y., Ren G., Feng B., Liu Z. (2013). Thioredoxin-like Protein 2 Is Overexpressed in Colon Cancer and Promotes Cancer Cell Metastasis by Interaction with Ran. Antioxid. Redox Signal..

[295] Li B., Chen M., Lu M., Xin-Xiang J., Meng-Xiong P., Jun-Wu M. (2018). Glutaredoxin 3 Promotes Migration and Invasion via the Notch Signalling Pathway in Oral Squamous Cell Carcinoma. Free Radic. Res..

[296] Qu Y., Wang J., Ray P.S., Guo H., Huang J., Shin-Sim M., Bukoye B.A., Liu B., Lee A.V., Lin X. (2011). Thioredoxin-like 2 Regulates Human Cancer Cell Growth and Metastasis via Redox Homeostasis and NF-ΚB Signaling. J. Clin. Investig..

[297] Somwar R., Erdjument-Bromage H., Larsson E., Shum D., Lockwood W.W., Yang G., Sander C., Ouerfelli O., Tempst P.J., Djaballah H. (2011). Superoxide Dismutase 1 (SOD1) Is a Target for a Small Molecule Identified in a Screen for Inhibitors of the Growth of Lung Adenocarcinoma Cell Lines. Proc. Natl. Acad. Sci. USA.

[298] Wang X., Zhang H., Sapio R., Yang J., Wong J., Zhang X., Guo J.Y., Pine S., Van Remmen H., Li H. (2021). SOD1 Regulates Ribosome Biogenesis in KRAS Mutant Non-Small Cell Lung Cancer. Nat. Commun..

[299] Song I.-S., Jeong Y.J., Jeong S.H., Heo H.J., Kim H.K., Bae K.B., Park Y.-H., Kim S.U., Kim J.-M., Kim N. (2015). FOXM1-Induced PRX3 Regulates Stemness and Survival of Colon Cancer Cells via Maintenance of Mitochondrial Function. Gastroenterology.

[300] Liu Z., Hu Y., Liang H., Sun Z., Feng S., Deng H. (2016). Silencing PRDX3 Inhibits Growth and Promotes Invasion and Extracellular Matrix Degradation in Hepatocellular Carcinoma Cells. J. Proteome Res..

[301] Cheng Y., Xu T., Li S., Ruan H. (2019). GPX1, a Biomarker for the Diagnosis and Prognosis of Kidney Cancer, Promotes the Progression of Kidney Cancer. Aging.

[302] Lv X., Yu H., Zhang Q., Huang Y., Hong X., Yu T., Lan H., Mei C., Zhang W., Luo H. (2020). SRXN1 Stimulates Hepatocellular Carcinoma Tumorigenesis and Metastasis through Modulating ROS/P65/BTG2 Signalling. J. Cell. Mol. Med..

[303] Jiang H., Wu L., Chen J., Mishra M., Chawsheen H.A., Zhu H., Wei Q. (2015). Sulfiredoxin Promotes Colorectal Cancer Cell Invasion and Metastasis through a Novel Mechanism of Enhancing EGFR Signaling. Mol. Cancer Res..

[304] Lee D., Xu I.M.-J., Chiu D.K.-C., Leibold J., Tse A.P.-W., Bao M.H.-R., Yuen V.W.-H., Chan C.Y.-K., Lai R.K.-H., Chin D.W.-C. (2019). Induction of Oxidative Stress Through Inhibition of Thioredoxin Reductase 1 Is an Effective Therapeutic Approach for Hepatocellular Carcinoma. Hepatology.

[305] Bu L., Li W., Ming Z., Shi J., Fang P., Yang S. (2017). Inhibition of TrxR2 Suppressed NSCLC Cell Proliferation, Metabolism and Induced Cell Apoptosis through Decreasing Antioxidant Activity. Life Sci..

[306] Bu L., Tian Y., Wen H., Jia W., Yang S. (2021). MiR-195-5p Exerts Tumor-Suppressive Functions in Human Lung Cancer Cells through Targeting TrxR2. Acta Biochim. Biophys. Sin..

[307] Chen H., Xu L., Shan Z., Chen S., Hu H. (2020). GPX8 Is Transcriptionally Regulated by FOXC1 and Promotes the Growth of Gastric Cancer Cells through Activating the Wnt Signaling Pathway. Cancer Cell Int..

[308] Yin X., Zhang P., Xia N., Wu S., Liu B., Weng L., Shang M. (2022). GPx8 Regulates Apoptosis and Autophagy in Esophageal Squamous Cell Carcinoma through the IRE1/JNK Pathway. Cell. Signal..

[309] Li S., Zhuang Z., Wu T., Lin J.-C., Liu Z.-X., Zhou L.-F., Dai T., Lu L., Ju H.-Q. (2018). Nicotinamide Nucleotide Transhydrogenase-Mediated Redox Homeostasis Promotes Tumor Growth and Metastasis in Gastric Cancer. Redox Biol..

[310] Kim B., Kim Y.S., Ahn H.-M., Lee H.J., Jung M.K., Jeong H.Y., Choi D.K., Lee J.H., Lee S.-R., Kim J.M. (2017). Peroxiredoxin 5 Overexpression Enhances Tumorigenicity and Correlates with Poor Prognosis in Gastric Cancer. Int. J. Oncol..

[311] Naiki T., Naiki-Ito A., Asamoto M., Kawai N., Tozawa K., Etani T., Sato S., Suzuki S., Shirai T., Kohri K. (2014). GPX2 Overexpression Is Involved in Cell Proliferation and Prognosis of Castration-Resistant Prostate Cancer. Carcinogenesis.

[312] Zhu L., Shen Y., Sun W. (2017). Paraoxonase 3 Promotes Cell Proliferation and Metastasis by PI3K/Akt in Oral Squamous Cell Carcinoma. Biomed. Pharmacother..

[313] Fumarola S., Cecati M., Sartini D., Ferretti G., Milanese G., Galosi A.B., Pozzi V., Campagna R., Morresi C., Emanuelli M. (2020). Bladder Cancer Chemosensitivity Is Affected by Paraoxonase-2 Expression. Antioxidants.

[314] Nagarajan A., Dogra S.K., Sun L., Gandotra N., Ho T., Cai G., Cline G., Kumar P., Cowles R.A., Wajapeyee N. (2017). Paraoxonase 2 Facilitates Pancreatic Cancer Growth and Metastasis by Stimulating GLUT1-Mediated Glucose Transport. Mol. Cell.

[315] Wang X., Xu G., Zhang J., Wang S., Ji M., Mo L., Zhu M., Li J., Zhou G., Lu J. (2019). The Clinical and Prognostic Significance of Paraoxonase-2 in Gastric Cancer Patients: Immunohistochemical Analysis. Hum. Cell.

[316] Pan L., Hong C., Chan L.N., Xiao G., Malvi P., Robinson M.E., Geng H., Reddy S.T., Lee J., Khairnar V. (2021). PON2 Subverts Metabolic Gatekeeper Functions in B Cells to Promote Leukemogenesis. Proc. Natl. Acad. Sci. USA.

[317] Zhao Y., Xue Y., Oberley T.D., Kiningham K.K., Lin S.M., Yen H.C., Majima H., Hines J., St Clair D. (2001). Overexpression of Manganese Superoxide Dismutase Suppresses Tumor Formation by Modulation of Activator Protein-1 Signaling in a Multistage Skin Carcinogenesis Model. Cancer Res..

[318] Zhang Z., Pratheeshkumar P., Budhraja A., Son Y.-O., Kim D., Shi X. (2015). Role of Reactive Oxygen Species in Arsenic-Induced Transformation of Human Lung Bronchial Epithelial (BEAS-2B) Cells. Biochem. Biophys. Res. Commun..

[319] Son Y.-O., Wang L., Poyil P., Budhraja A., Hitron J.A., Zhang Z., Lee J.-C., Shi X. (2012). Cadmium Induces Carcinogenesis in BEAS-2B Cells through ROS-Dependent Activation of PI3K/AKT/GSK-3β/β-Catenin Signaling. Toxicol. Appl. Pharm..

[320] Pratheeshkumar P., Son Y.-O., Divya S.P., Roy R.V., Hitron J.A., Wang L., Kim D., Dai J., Asha P., Zhang Z. (2014). Luteolin Inhibits Cr(VI)-Induced Malignant Cell Transformation of Human Lung Epithelial Cells by Targeting ROS Mediated Multiple Cell Signaling Pathways. Toxicol. Appl. Pharm..

[321] Wang X., Son Y.-O., Chang Q., Sun L., Hitron J.A., Budhraja A., Zhang Z., Ke Z., Chen F., Luo J. (2011). NADPH Oxidase Activation Is Required in Reactive Oxygen Species Generation and Cell Transformation Induced by Hexavalent Chromium. Toxicol. Sci..

[322] St. Clair D.K., Steven Wan X., Oberley T.D., Muse K.E., St. Clair W.H. (1992). Suppression of Radiation-Induced Neoplastic Transformation by Overexpression of Mitochondrial Superoxide Dismutase. Mol. Carcinog..

[323] Nakamura Y., Gindhart T.D., Winterstein D., Tomita I., Seed J.L., Colburn N.H. (1988). Early Superoxide Dismutase-Sensitive Event Promotes Neoplastic Transformation in Mouse Epidermal JB6 Cells. Carcinogenesis.

[324] Jia X., Guan B., Liao J., Hu X., Fan Y., Li J., Zhao H., Huang Q., Ma Z., Zhu X. (2019). Down-Regulation of GCLC Is Involved in Microcystin-LR-Induced Malignant Transformation of Human Liver Cells. Toxicology.

[325] Guo X., Noguchi H., Ishii N., Homma T., Hamada T., Hiraki T., Zhang J., Matsuo K., Yokoyama S., Ishibashi H. (2019). The Association of Peroxiredoxin 4 with the Initiation and Progression of Hepatocellular Carcinoma. Antioxid. Redox Signal..

[326] Yi Z., Jiang L., Zhao L., Zhou M., Ni Y., Yang Y., Yang H., Yang L., Zhang Q., Kuang Y. (2019). Glutathione Peroxidase 3 (GPX3) Suppresses the Growth of Melanoma Cells through Reactive Oxygen Species (ROS)-Dependent Stabilization of Hypoxia-Inducible Factor 1-α and 2-α. J. Cell. Biochem..

[327] An B.C., Choi Y.-D., Oh I.-J., Kim J.H., Park J.-I., Lee S.-W. (2018). GPx3-Mediated Redox Signaling Arrests the Cell Cycle and Acts as a Tumor Suppressor in Lung Cancer Cell Lines. PLoS ONE.

[328] Qi X., Ng K.T.P., Lian Q.Z., Liu X.B., Li C.X., Geng W., Ling C.C., Ma Y.Y., Yeung W.H., Tu W.W. (2014). Clinical Significance and Therapeutic Value of Glutathione Peroxidase 3 (GPx3) in Hepatocellular Carcinoma. Oncotarget.

[329] Yu Y.P., Yu G., Tseng G., Cieply K., Nelson J., Defrances M., Zarnegar R., Michalopoulos G., Luo J.-H. (2007). Glutathione Peroxidase 3, Deleted or Methylated in Prostate Cancer, Suppresses Prostate Cancer Growth and Metastasis. Cancer Res..

[330] Cai M., Sikong Y., Wang Q., Zhu S., Pang F., Cui X. (2019). Gpx3 Prevents Migration and Invasion in Gastric Cancer by Targeting NFκB/Wnt5a/JNK Signaling. Int. J. Clin. Exp. Pathol..

[331] Zhu X., Wang J., Li L., Deng L., Wang J., Liu L., Zeng R., Wang Q., Zheng Y. (2018). GPX3 Suppresses Tumor Migration and Invasion via the FAK/AKT Pathway in Esophageal Squamous Cell Carcinoma. Am. J. Transl. Res..

[332] Peng D.-F., Hu T.-L., Schneider B.G., Chen Z., Xu Z.-K., El-Rifai W. (2012). Silencing of Glutathione Peroxidase 3 through DNA Hypermethylation Is Associated with Lymph Node Metastasis in Gastric Carcinomas. PLoS ONE.

[333] Lou W., Ding B., Wang S., Fu P. (2020). Overexpression of GPX3, a Potential Biomarker for Diagnosis and Prognosis of Breast Cancer, Inhibits Progression of Breast Cancer Cells in Vitro. Cancer Cell. Int..

[334] Hong S.H., Min C., Jun Y., Lee D.J., Kim S.H., Park J.H., Cheong J.H., Park Y.J., Kim S.-Y., Lee S. (2018). Silencing of Peroxiredoxin II by Promoter Methylation Is Necessary for the Survival and Migration of Gastric Cancer Cells. Exp. Mol. Med..

[335] Feng J., Fu Z., Guo J., Lu W., Wen K., Chen W., Wang H., Wei J., Zhang S. (2014). Overexpression of Peroxiredoxin 2 Inhibits TGF-Β1-Induced Epithelial-Mesenchymal Transition and Cell Migration in Colorectal Cancer. Mol. Med. Rep..

[336] Xiao H., Yang T., Yan L., Feng J., Huang B., Jiang Y. (2020). PRDX1 Is a Tumor Suppressor for Nasopharyngeal Carcinoma by Inhibiting PI3K/AKT/TRAF1 Signaling. Onco Targets Ther..

[337] Wang Y., Liu M., Yang P., Peng H. (2018). Peroxiredoxin 1 (PRDX1) Suppresses Progressions and Metastasis of Osteosarcoma and Fibrosarcoma of Bone. Med. Sci. Monit..

[338] Mougiakakos D., Okita R., Ando T., Dürr C., Gadiot J., Ichikawa J., Zeiser R., Blank C., Johansson C.C., Kiessling R. (2012). High Expression of GCLC Is Associated with Malignant Melanoma of Low Oxidative Phenotype and Predicts a Better Prognosis. J. Mol. Med..

[339] Ren Z., Liang H., Galbo P.M., Dharmaratne M., Kulkarni A.S., Fard A.T., Aoun M.L., Martinez-Lopez N., Suyama K., Benard O. (2022). Redox Signaling by Glutathione Peroxidase 2 Links Vascular Modulation to Metabolic Plasticity of Breast Cancer. Proc. Natl. Acad. Sci. USA.

[340] Heirman I., Ginneberge D., Brigelius-Flohé R., Hendrickx N., Agostinis P., Brouckaert P., Rottiers P., Grooten J. (2006). Blocking Tumor Cell Eicosanoid Synthesis by GPx4 Impedes Tumor Growth and Malignancy. Free Radic. Biol. Med..

[341] Devarajan A., Su F., Grijalva V., Yalamanchi M., Yalamanchi A., Gao F., Trost H., Nwokedi J., Farias-Eisner G., Farias-Eisner R. (2018). Paraoxonase 2 Overexpression Inhibits Tumor Development in a Mouse Model of Ovarian Cancer. Cell Death Dis..

[342] Cai J., Yuan S.-X., Yang F., Tao Q.-F., Yang Y., Xu Q.-G., Wang Z.-G., Yu J., Lin K.-Y., Wang Z.-Y. (2016). Paraoxonase 3 Inhibits Cell Proliferation and Serves as a Prognostic Predictor in Hepatocellular Carcinoma. Oncotarget.

[343] Huang D., Wang Y., He Y., Wang G., Wang W., Han X., Sun Y., Lin L., Shan B., Shen G. (2018). Paraoxonase 3 Is Involved in the Multi-Drug Resistance of Esophageal Cancer. Cancer Cell Int..

[344] Chen B., Rao X., House M.G., Nephew K.P., Cullen K.J., Guo Z. (2011). GPx3 Promoter Hypermethylation Is a Frequent Event in Human Cancer and Is Associated with Tumorigenesis and Chemotherapy Response. Cancer Lett..

[345] Peng D.F., Razvi M., Chen H., Washington K., Roessner A., Schneider-Stock R., El-Rifai W. (2009). DNA Hypermethylation Regulates the Expression of Members of the Mu-Class Glutathione S-Transferases and Glutathione Peroxidases in Barrett’s Adenocarcinoma. Gut.

[346] Zhang X., Yang J.-J., Kim Y.S., Kim K.-Y., Ahn W.S., Yang S. (2010). An 8-Gene Signature, Including Methylated and down-Regulated Glutathione Peroxidase 3, of Gastric Cancer. Int. J. Oncol..

[347] de los Santos-Jiménez J., Campos-Sandoval J.A., Márquez-Torres C., Urbano-Polo N., Brøndegaard D., Martín-Rufián M., Lobo C., Peñalver A., Gómez-García M.C., Martín-Campos J. (2021). Glutaminase Isoforms Expression Switches MicroRNA Levels and Oxidative Status in Glioblastoma Cells. J. Biomed. Sci..

[348] Zhao Z., Song J., Tang B., Fang S., Zhang D., Zheng L., Wu F., Gao Y., Chen C., Hu X. (2020). CircSOD2 Induced Epigenetic Alteration Drives Hepatocellular Carcinoma Progression through Activating JAK2/STAT3 Signaling Pathway. J. Exp. Clin. Cancer Res..

[349] Bacchetti T., Salvolini E., Pompei V., Campagna R., Molinelli E., Brisigotti V., Togni L., Lucarini G., Sartini D., Campanati A. (2021). Paraoxonase-2: A Potential Biomarker for Skin Cancer Aggressiveness. Eur. J. Clin. Investig..

[350] Bacchetti T., Ferretti G., Sahebkar A. (2019). The Role of Paraoxonase in Cancer. Semin. Cancer Biol..

[351] Gandhi G.R., Leão G.C.d.S., Calisto V.K.d.S., Vasconcelos A.B.S., Almeida M.L.D., Quintans J.d.S.S., Barreto E., Narain N., Júnior L.J.Q., Gurgel R.Q. (2020). Modulation of Interleukin Expression by Medicinal Plants and Their Secondary Metabolites: A Systematic Review on Anti-Asthmatic and Immunopharmacological Mechanisms. Phytomedicine.

[352] Sitarek P., Merecz-Sadowska A., Kowalczyk T., Wieczfinska J., Zajdel R., Śliwiński T. (2020). Potential Synergistic Action of Bioactive Compounds from Plant Extracts against Skin Infecting Microorganisms. Int. J. Mol. Sci..

[353] Hasanpour M., Iranshahy M., Iranshahi M. (2020). The Application of Metabolomics in Investigating Anti-Diabetic Activity of Medicinal Plants. Biomed. Pharmacother..

